# Systematic in vitro analysis of therapy resistance in glioblastoma cell lines by integration of clonogenic survival data with multi-level molecular data

**DOI:** 10.1186/s13014-023-02241-4

**Published:** 2023-03-11

**Authors:** Leon Emanuel Schnöller, Daniel Piehlmaier, Peter Weber, Nikko Brix, Daniel Felix Fleischmann, Alexander Edward Nieto, Martin Selmansberger, Theresa Heider, Julia Hess, Maximilian Niyazi, Claus Belka, Kirsten Lauber, Kristian Unger, Michael Orth

**Affiliations:** 1 Department of Radiation Oncology, University Hospital, LMU München, Marchioninistrasse 15, 81377 Munich, Germany; 2 Research Unit Radiation Cytogenetics (ZYTO), Helmholtz Center Munich, German Research Center for Environmental Health GmbH, 85764 Neuherberg, Germany; 3 Clinical Cooperation Group ‘Personalized Radiotherapy in Head and Neck Cancer’ Helmholtz Center Munich, German Research Center for Environmental Health GmbH, Neuherberg, Germany; 4 German Cancer Consortium (DKTK), Munich, Germany; 5 German Cancer Research Center (DKFZ), Heidelberg, Germany; 6 Bavarian Cancer Research Center (BKFZ), Munich, Germany

**Keywords:** Glioblastoma, Therapy resistance, Multi-level molecular data, Correlation analysis

## Abstract

Despite intensive basic scientific, translational, and clinical efforts in the last decades, glioblastoma remains a devastating disease with a highly dismal prognosis. Apart from the implementation of temozolomide into the clinical routine, novel treatment approaches have largely failed, emphasizing the need for systematic examination of glioblastoma therapy resistance in order to identify major drivers and thus, potential vulnerabilities for therapeutic intervention. Recently, we provided proof-of-concept for the systematic identification of combined modality radiochemotherapy treatment vulnerabilities via integration of clonogenic survival data upon radio(chemo)therapy with low-density transcriptomic profiling data in a panel of established human glioblastoma cell lines. Here, we expand this approach to multiple molecular levels, including genomic copy number, spectral karyotyping, DNA methylation, and transcriptome data. Correlation of transcriptome data with inherent therapy resistance on the single gene level yielded several candidates that were so far underappreciated in this context and for which clinically approved drugs are readily available, such as the androgen receptor (AR). Gene set enrichment analyses confirmed these results, and identified additional gene sets, including reactive oxygen species detoxification, mammalian target of rapamycin complex 1 (MTORC1) signaling, and ferroptosis/autophagy-related regulatory circuits to be associated with inherent therapy resistance in glioblastoma cells. To identify pharmacologically accessible genes within those gene sets, leading edge analyses were performed yielding candidates with functions in thioredoxin/peroxiredoxin metabolism, glutathione synthesis, chaperoning of proteins, prolyl hydroxylation, proteasome function, and DNA synthesis/repair. Our study thus confirms previously nominated targets for mechanism-based multi-modal glioblastoma therapy, provides proof-of-concept for this workflow of multi-level data integration, and identifies novel candidates for which pharmacological inhibitors are readily available and whose targeting in combination with radio(chemo)therapy deserves further examination. In addition, our study also reveals that the presented workflow requires mRNA expression data, rather than genomic copy number or DNA methylation data, since no stringent correlation between these data levels could be observed. Finally, the data sets generated in the present study, including functional and multi-level molecular data of commonly used glioblastoma cell lines, represent a valuable toolbox for other researchers in the field of glioblastoma therapy resistance.

**Supplementary Information:**

The online version contains supplementary material available at 10.1186/s13014-023-02241-4.

## Introduction

Glioblastoma, despite immense efforts in preclinical, translational, and clinical research during the last decades still remains a daunting disease with highly dismal prognosis [1]. Fractionated radio(chemo)therapy with 30 fractions of 2 Gy and concomitant administration of DNA-alkylating temozolomide (TMZ) in definitive or adjuvant settings, followed by TMZ-based maintenance therapy remains the standard of care [2, 3]. However, glioblastoma is well-known for its high degree of inherent therapy resistance, both to ionizing radiation (IR) and TMZ, resulting in frequent treatment failure and early recurrence [4]. Thus, a more detailed understanding of the biological and molecular mechanisms underlying glioblastoma therapy resistance is needed in order to open new perspectives for molecularly targeted therapy and to improve disease prognosis [5, 6].

Based on our recent proof-of-concept study [7], we here present a systematic approach which integrates inherent resistance data of commonly used human glioblastoma cell lines to IR and TMZ with multi-level molecular data of those cell lines obtained under treatment-naive conditions, including spectral karyotyping (SKY FISH), array comparative genomic hybridization (aCGH), array-based DNA methylation, and transcriptomic (mRNA microarray) analyses. By integrating these data with scores of inherent therapy resistance as extracted by dimensionality reduction of clonogenic survival data ([7, 8], Additional file 1: Table S1), and subsequent gene set enrichment analysis (GSEA) [9], we could identify several candidate networks and signaling circuits which are associated with inherent treatment resistance of glioblastoma cells and can be readily targeted by clinically approved or at least clinically trialed drugs. Among these were pathways involved in homeostasis of reactive oxygen species (ROS), mammalian target of rapamycin complex 1 (mTORC1) signaling, and androgen receptor (AR) signaling [10, 11]. Taken together, we present a novel integrative approach for the systematic identification of therapeutic vulnerabilities (not only) in glioblastoma as well as potential candidates whose targeting in conjunction with radiotherapy and/or TMZ may help to break glioblastoma therapy resistance.

## Methods

### Cell culture

The human glioblastoma cell lines A172, LN18, LN229, T98G, U87 [12], U138, and U251 were purchased from the American Type Culture Collection (ATCC, Manassas, VA, USA), or from Cell Lines Service GmbH (CLS, Eppelheim, Germany), and confirmed for identity by short tandem repeat (STR) typing according to the standards of the ATCC and the American National Standards Institute (ANSI, New York, NY, USA) of 2011 (ANSI/ATCC ASN-0002–2011) [13]. All cell lines were cultured in Dulbecco’s Modified Eagle Medium (D-MEM) supplemented with 10% (v/v) heat-inactivated fetal calf serum (FCS), 100 U/ml penicillin, and 0.1 mg/mL streptomycin (all from Thermo Scientific, Schwerte, Germany) at 37 °C and 7.5% CO_2_. Cells were kept at low passage numbers (≤ 10 passages), and were regularly tested by MycoAlert assay (Lonza, Basel, Switzerland) to be free of mycoplasma contamination.

### Determination of therapy resistance scores

Resistance to therapy was determined by clonogenic survival assays as described [7]. In brief, cells were seeded into 6-well plates and incubated for 4 h in order to adhere. Cells were irradiated at the indicated doses in single-shot or in intervals of 24 h (fractionated mode), and colony formation was allowed for up to 12 d. In case of TMZ treatment, cells were incubated with TMZ at the indicated doses for 24 h, medium was changed, and colony formation was performed in TMZ-free medium. For the combination treatment, cells were exposed to 5 µM TMZ for 24 h, irradiated at the indicated doses, and incubated in TMZ-free medium for colony formation. Colonies were fixed with 80% ethanol, stained with 0.8% methylene blue (both from Merck Millipore, Darmstadt, Germany), and counted with a Stemi 305 stereomicroscope (Carl Zeiss, Oberkochen, Germany) as described [7, 14]. Percentages of colony forming cells were calculated and normalized to the respective plating efficiencies at approximately matched colony numbers. Resistance scores were extracted by principal component analysis (PCA) as scores of the first principal component (PC1) [8].

### Spectral karyotyping (SKY FISH)

For spectral karyotyping (SKY FISH) analyses, cells at 80% confluency were accumulated in M-phase by treatment with 0.1 µg/ml colcemid (Roche Diagnostics, Basel, Switzerland) for 3 h. Cells were then harvested with TrypLE Express (Thermo Scientific), and incubated in 4.0% (w/v) potassium chloride for 45 min at 37 °C. Cells were fixed with fixative (methanol and glacial acetic acid at 3:1 ratio, both from Merck Millipore) for 45 min at 4 °C, washed, and resuspended in fixative. Cell suspensions were dripped onto microscope slides, and hybridization was performed using the denatured SKY-probe mixture kit SkyPAINT DNA (Applied Spectral Imaging, Carlsbad, CA, USA) as previously described [15]. For staining, slides were incubated with anti-digoxigenin (Roche), avidin-Cy5, and avidin-Cy5.5 antibodies (Biomol, Hamburg, Germany), and counterstained with 0.1% (w/v) 4’,6-diamidino-2-phenylindole (DAPI, Sigma-Aldrich, Taufkirchen, Germany). Slides were supplemented with Vectashield mounting solution (Vector Laboratories, Burlingame, CA, USA), and spectral imaging was performed with a Zeiss Axioplan 2 fluorescence microscope (Carl Zeiss, Oberkochen, Germany) equipped with a SpectraCube device and SkyView software (both from Applied Spectral Imaging). Description of karyotypes was performed according to the international system of human cytogenetic nomenclature (ISCN, edition 2013) [16].

### Global gene expression microarrays

To analyze global mRNA expression levels in glioblastoma cells, gene expression microarray analysis was performed. In brief, total RNA was extracted from cells using the Qiagen Allprep DNA/RNA mini kit (Qiagen). RNA concentration was determined with a Nanodrop ND-1000 spectrophotometer (Thermo Scientific), and RNA quality was assessed by a total RNA 6000 nano chip assay performed on an Agilent 2100 Bioanalyzer (Agilent Technologies, Santa Clara, CA, USA). To obtain global mRNA expression data, 50 ng of total RNA was subjected to Agilent SurePrint G3 human 8 × 60 k V2 microarray analysis (AMADID 039,494, Agilent Technologies). RNA from untransformed human astrocytes (Provitro AG, Berlin, Germany) served as reference. Results were collected using the Agilent feature extraction software (version 10.7, Agilent Technologies), and exported as text files. Assessment of data quality, filtering, and data processing were performed with the Bioconductor R packages Limma and Agi4 × 44PreProcess as previously described [17], and data analysis was performed with R. The obtained data are available at Gene Expression Omnibus (GEO, super set accession number: GSE119637) and under the link https://www.ncbi.nih.gov/geo/query/acc.cgi?acc=GSE119637 using the token ‘sfqnocumrvofrmv’.

### Array comparative genomic hybridization (aCGH)

To identify genomic copy number alterations (CNAs) in glioblastoma cells, array comparative genomic hybridization (aCGH) analyses were performed as previously described [18]. In brief, DNA was extracted from cells by Qiagen Allprep DNA/RNA mini kit (Qiagen), concentration and quality of DNA were assessed with a Nanodrop ND-1000 spectrophotometer (Thermo Scientific), and 120 ng DNA was fluorescently labelled using the CYTAG SuperCGH labelling kit (Enzo Life Sciences, New York, NY, USA). After removing free nucleotides using Microcon YM-30 columns (Merck Millipore), labelled DNA was subjected to oligonucleotide-based high-resolution SurePrint G3 Human 60 k CGH microarray analysis (AMADID 021,924, Agilent Technologies). Microarrays were scanned with a G2505C SureScan microarray scanning system (Agilent Technologies), raw data were extracted using the Agilent feature extraction software (version 10.7, Agilent Technologies), and CNA regions were identified using the Bioconductor R packages CGHcall and CGHregions [18, 19]. A compilation of all molecular data collected in this study is deposited as an Excel file in the Additional file 2.

### Array-based DNA methylation analyses

Array-based DNA methylation analyses were performed as previously described [7]. In brief, DNA was extracted by Qiagen Allprep DNA/RNA mini kit (Qiagen), concentration and quality of DNA were assessed with a Nanodrop ND-1000 spectrophotometer (Thermo Scientific), and 500 ng DNA was subjected to hybridization on an Infinium EPIC methylation array (Illumina, San Diego, CA; USA). The arrays were scanned, idat files were imported in R using the minfi package [20], and processed in accordance to the Illumina BeadStudio data analysis workflow (Illumina). Beta values were used for downstream hierarchical clustering analysis along with the beta values retrieved for glioblastoma samples from TCGA database https://www.cancer.gov/tcga [21, 22].

### Comparison of transcriptome and methylome profiles of glioblastoma cell lines with those from glioblastoma and lower-grade glioma (LGG) patient samples

**Fig. 1:**
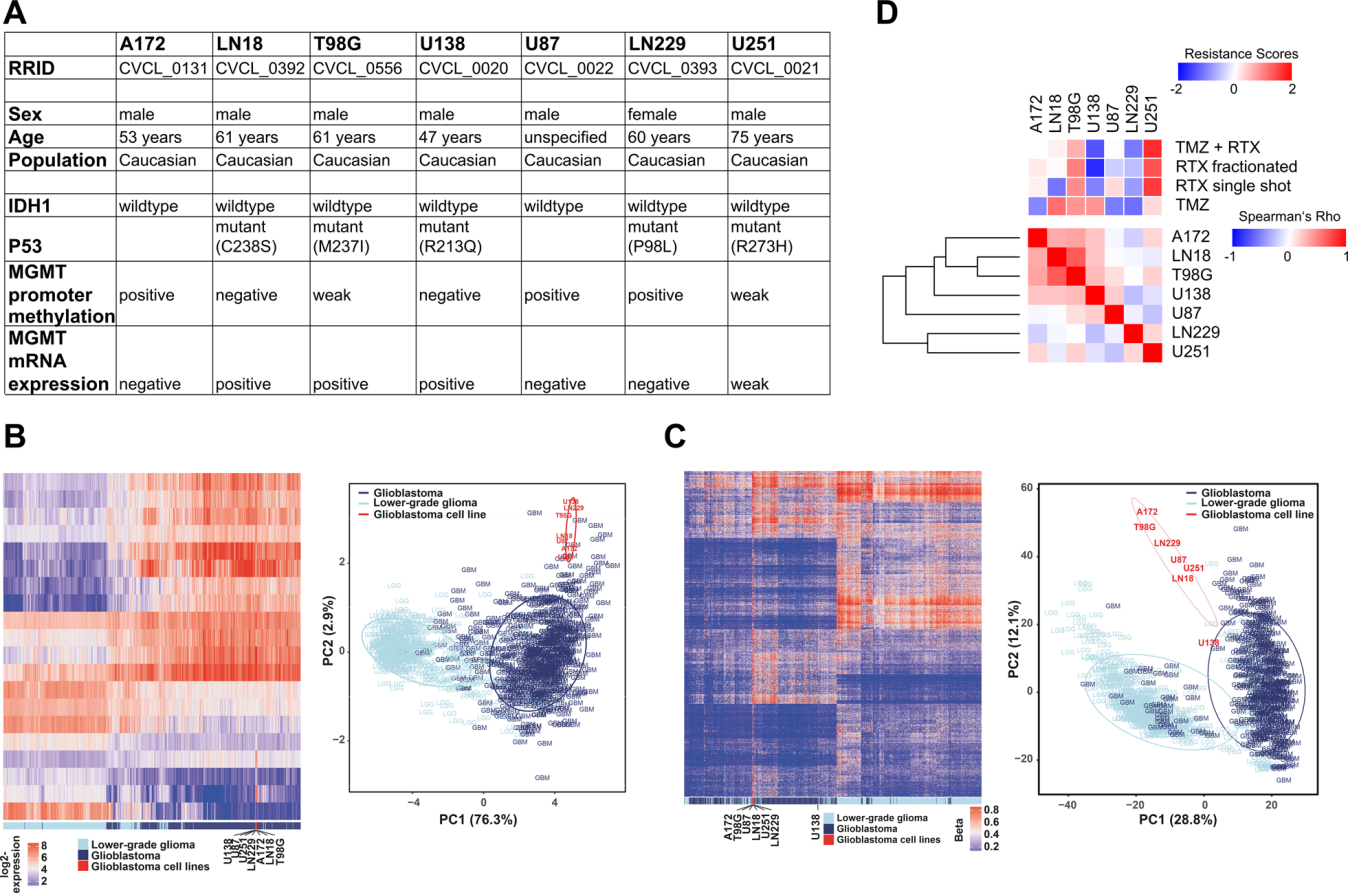
Sample-to-sample correlation of mRNA expression of 100 most differently expressed genes in a human glioblastoma cell line panel does not match with inherent therapy resistance** a** Tabular presentation of characteristics of the human glioblastoma cell lines as obtained from the Cellosaurus database (https://web.expasy.org/cellosaurus/). **b** Unsupervised hierarchical clustering and principal component analysis (PCA) of mRNA expression levels of top 20 genes differently expressed between glioblastoma and low-grade glioma (LGG) patient samples (data from the TCGA database (https://tcga-data.nci.nih.gov/docs/publications/lgggbm_2015/), clustering method ward.D and euclidean distance measure) in 560 glioblastoma and 463 LGG patient samples, and in glioblastoma cell lines. **c** Unsupervised hierarchical clustering and PCA of G-CIMP signatures for hypermethylation phenotypes in 410 glioblastoma and 516 LGG patient samples (data from the TCGA database), and in glioblastoma cell lines. **d** Sample-to-sample correlation analysis of mRNA expression of 100 genes with highest intra-panel variation in expression in human glioblastoma cell lines. Expression values were determined by global gene expression microarray analysis. PCA-derived scores of inherent resistance (PC1s as described in [7, 8]) to single-shot radiotherapy (RTX), fractionated RTX, TMZ, and TMZ + single-shot RTX are depicted on top by unsupervised hierarchical clustering

Transcriptome profiles and methylome beta values from glioblastoma and LGG patient samples were retrieved from TCGA (https://tcga-data.nci.nih.gov/docs/publications/lgggbm_2015/) [21, 22]. Transcriptome profiles of 560 glioblastoma and 463 LGG patients were corrected for putative batch effects and z-scaled per gene before combination with the transcriptomic data obtained from the cell lines in a common gene expression matrix. The top 20 differently expressed genes between glioblastoma and LGG samples were analyzed by hierarchical clustering (Fig. 1b, clustering method ward.D and euclidean distance measure), and principal component analysis (PCA).

For DNA methylation, the profiles of 410 glioblastoma and 516 LGG patients were included, and a merged beta value matrix including the methylation profiles of glioblastoma and LGG patients as well as those of the glioblastoma cell lines matching the CpG island methylator phenotype (CIMP) signature by Ceccarelli et al. [21] was generated. The methylation profiles were subjected to hierarchical clustering analysis (Fig. 1c, clustering method ward.D and euclidean distance measure), and analyzed by PCA.

### Molecular subtyping based on cytogenetic and transcriptomic data

**Fig. 2:**
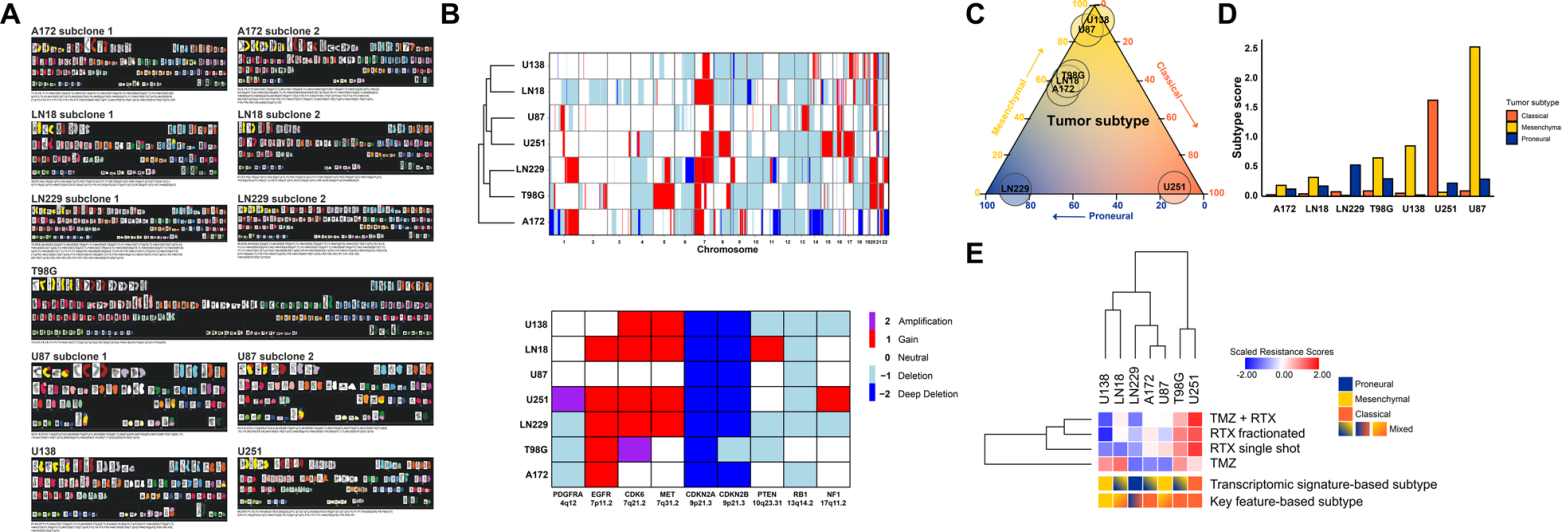
Molecular subtyping of human glioblastoma cell lines. **a** Spectral karyotyping (SKY FISH) analyses of human glioblastoma cell lines. SKY FISH analyses were performed as previously described [15]. Karyotypes were described according to the international system of human cytogenetic nomenclature (ISCN, edition 2013) [16]. For cell lines A172, LN18, LN229, and U87, two distinct cytogenetic subclones are shown each. **b** Array comparative genomic hybridization (aCGH) analysis of glioblastoma cell lines. Hierarchical clustering of genomic copy number calls of chromosomes 1–22 in glioblastoma cell lines (top), and copy number status of gene loci with known association to glioblastoma subtypes (bottom). Copy number gains (up to 4 copies) are depicted by red bars, copy number amplifications (> 4 copies) by purple bars, and copy number losses by light blue (1 copy), and dark blue (complete loss), respectively. **c** Ternary plot of molecular subtyping of human glioblastoma cell lines on basis of mRNA expression of subtype-related signature genes (according to Wang et al. [23]), obtained by ssGSEA. The proneural subtype is depicted in blue, the classical subtype is depicted in orange, and the mesenchymal subtype is depicted in yellow. Values for subtypes were scaled to sum of 100% per cell line. **d** Bar plots of molecular subtype scores. **e** Graphical presentation of molecular subtypes of human glioblastoma cell lines as revealed by transcriptomic signature-based (top) and key feature-based subtyping (bottom), respectively. Scores of inherent therapy resistance are depicted by unsupervised hierarchical clustering

Molecular subtyping was performed via different approaches. For calculation of molecular subtype scores (Additional file 1: Table S3), the data derived from cytogenetic characterization (SKY FISH) and aCGH analyses together with the expression data of relevant driver genes were utilized. All molecular subtype features of a cell line were summed up before being divided by the total number of features (Fig. 2c). Alternatively, molecular subtyping was performed by the single-sample gene set enrichment analysis (ssGSEA) algorithm provided as an R-package by Wang et al. [23], and using the reduced transcriptomic signatures from Verhaak et al. [24] (Fig. 2d). The negative log10 values of the resulting p-values were used as molecular subtype scores, and according to the maximum value the molecular subtype was assigned.

### Integration of therapy resistance with microarray expression data

Integration of therapy resistance data with microarray transcriptome data was performed via different alternative approaches. On single gene level, log2 expression values obtained by microarray analyses were subjected to Pearson's correlation with resistance scores as extracted from clonogenic survival data via PCA (Additional file 1: Table S1 and [7, 8]). Similarly, but with focus on cancer-related genes, only members of the cancer gene consensus (CGC), a library of genes stored in the catalogue of somatic mutations in cancer (COSMIC) [25] were used for correlation analyses. Thirdly, integration on gene set level was performed by ranking genes in accordance to their respective correlation coefficients with therapy resistance scores followed by pre-ranked gene set enrichment analyses (GSEAs) [9]. Leading edge genes were visualized by functional interaction networks in Cytoscape (Cytoscape Consortium, San Diego, CA, USA) [26]. Lastly, integration on gene set level was alternatively performed via an inverse workflow in which the complexity of microarray expression data was first reduced by gene set variation analysis (GSVA) [26] in single samples, followed by Pearson's correlation analyses with therapy resistance scores.

## Results

### Major differences in global mRNA expression patterns do not match with scores of inherent therapy resistance in a panel of human glioblastoma cell lines

The high degree of therapy resistance, the dismal prognosis, and the persisting lack of prognostic and/or predictive factors in glioblastoma demand for the identification of novel stratification markers for treatment responses on one hand, and potential vulnerabilities for combined modality strategies on the other [6, 27, 28]. Very recently, we published a proof-of-concept screen in which scores of inherent treatment resistance of glioblastoma cells, both to IR and TMZ (Additional file 1: Table S1), were correlated with basal mRNA expression levels of genes related to the DNA damage response (DDR)—a promising target to undermine therapy resistance in glioblastoma [7]. We were able to identify several DDR genes whose mRNA expression levels showed significant positive correlation with inherent therapy resistance, and pharmacological interference with the function of some of the corresponding gene products using specific inhibitors indeed resulted in sensitization of resistant glioblastoma cells to IR or TMZ treatment, respectively [7].

In the present study, we expanded this workflow to the global transcriptomic level and performed gene expression and DNA methylation microarray analyses using the same cell line panel as before (Fig. 1a, [7]). We first employed the transcriptome/methylome profiles of the cell lines to analyze their relatedness to clinical tumor samples, either from glioblastoma or from lower-grade glioma (LGG) patients as available from TCGA (Fig. 1b, c). All glioblastoma cell lines clustered closer to the glioblastoma patient samples than to the LGG samples, both on the transcriptome (Fig. 1b) and the methylome level (Fig. 1c). However, the cell lines were located at the periphery of the glioblastoma sample cluster, indicating a traceable but limited relatedness which may derive from the absence of non-tumor cells in the cell lines, adaptation processes in cell culture, or other reasons, respectively. We next examined whether the most striking transcriptomic differences across the cell line panel can be linked to inherent therapy resistances of glioblastoma cells. However, sample-to-sample correlation analyses on basis of the 100 most differentially expressed genes resulted in clusters of cell lines without obvious associations to therapy resistance (Fig. 1d). As an example, LN18 and T98G cells, the two most closely related cell lines according to sample-to-sample correlation of their expression profiles revealed strong differences in their respective levels of treatment resistance (Fig. 1d, and [7]) which was most obvious for regimens encompassing IR. Vice versa, cell lines with similar levels of treatment resistance, for instance A172 and U87, showed very far relatedness in sample-to-sample correlation of their gene expression profiles (Fig. 1d). Thus, inherent therapy resistance of glioblastoma cells cannot be directly linked to major differences in global mRNA expression patterns, at least in the cell line panel we analyzed here. Obviously, more systematic approaches integrating large-scale molecular (OMICs) data with functional data are needed [4].

### Multi-level cytogenetic and molecular characterization of human glioblastoma cell lines allows their classification into defined molecular subtypes

Several classification systems of glioblastoma on different molecular levels have been described [29]. Emerging consensus is the categorization according to genomic and transcriptomic features into three defined subtypes, termed the classical, the proneural, and the mesenchymal subtype [23, 24]. Model systems of these subtypes have been reported to exhibit marked differences in the response to therapy in vitro and in vivo [30, 31], and the mesenchymal subtype was found to be the most refractory. Intriguingly, strong phenotypic plasticity between subtypes could be observed [30, 31], and the transition from the proneural to the mesenchymal subtype was described to be an important driver of therapeutic failure [30–32]. Clinically, the mesenchymal subtype exhibits the most dismal and the proneural subtype the most benign prognosis, suggested to be driven by subtype-specific signaling pathways including DNA damage repair, cell cycle control, mesenchymal cell movement, mitogen-activated protein kinase (MAPK)/extracellular signal-regulated kinase (ERK) signaling, PI3K/AKT signaling, JAK/STAT signaling, and WNT pathways [23, 29, 33].

We therefore aimed to classify the glioblastoma cell lines of our panel according to these described subtypes. Firstly, we performed spectral karyotyping (SKY FISH) analyses (Fig. 2a and Additional file 1: Table S2). The karyotypes ranged from a near-diploid chromosomal content in U87 cells up to a near-hexaploid one found in T98G (Fig. 2a and Additional file 1: Table S2). We also noticed that some of the cell lines (A172, LN18, LN229, T98G, and U87) displayed different cytogenetic subclones (Fig. 2a and Additional file 1: Table S2) which is indicative for enhanced chromosomal instability (CIN) in these cell lines [34]. Increased CIN, in turn, is known to affect all major aspects of cancer pathogenicity including tumor progression, metastasis formation, and therapy resistance [35–37], and this also holds true for glioblastoma [38, 39]. Mechanistically, elevated CIN leads to gene copy number alterations (CNAs), which in case of affecting oncogenic driver genes can give rise to the aforementioned subtypes [40, 41]. Distinct CNAs were shown to be associated with different molecular subtypes [24, 42, 43], and we therefore performed array comparative genomic hybridization (aCGH) analyses (Fig. 2b and Additional file 1: Table S3). CNAs with documented association to the classical subtype, including amplification of 7p11.2 (EGFR), and focal deletions of 9p21.3 (CDKN2A) [24], were detected in most of the cell lines (Fig. 2b and Additional file 1: Table S3), whereas CNAs with association to the mesenchymal (loss of 17p11.2, NF1) or the proneural subtype (amplification of 4q12, PDGFRA) were only rarely detected. According to our transcriptomic analyses, driver genes of the mesenchymal subtype, such as TRADD, RELB, TNFRSF1A, and CASP1 [24, 42], were widely expressed. On the contrary, expression of genes related to the proneural subtype including NKX2-2, OLIG2, SOX2, and ERBB3 [24, 44–46] was only detected in one cell line given by LN229 (Additional file 1: Table S3), and genes linked to the classical subtype such as NOTCH3, NES, and SMO [47] were expressed heterogeneously across our panel. In synopsis, subtyping on the basis of cytogenetic and transcriptomic key features classified cell lines A172, T98G, and U251 as classical, and U138 as mesenchymal, but failed to deliver clear-cut classifications for cell lines LN18, LN229, and U87 (Fig. 2e and Additional file 1: Table S3).

Subtyping on the basis of transcriptomic signature genes according to Wang et al. [23] revealed a more heterogeneous pattern of subtypes across our cell line panel (Fig. 2c-e). For A172, LN18, and T98G no clear-cut classifications could be obtained (denoted as "mixed"). These cell lines were located on the proneural-mesenchymal axis (Fig. 2c), presumably reflecting the aforementioned plasticity in proneural-mesenchymal transition. For LN229, U87, U138, and U251, on the contrary, clear-cut classifications could indeed be achieved, classifying LN229 as proneural, U251 as classical, and U87 and U138 as resembling the prognostically challenging mesenchymal subtype (Fig. 2c-e). Reasons for the slight prevalence of this subtype among the panel could be excessive clonal selection and adaptation during initial establishment, and long-term cultivation of these cell lines.

### Integrating global gene expression data with clonogenic survival data as a strategy to identify new markers of glioblastoma therapy resistance

**Fig. 3:**
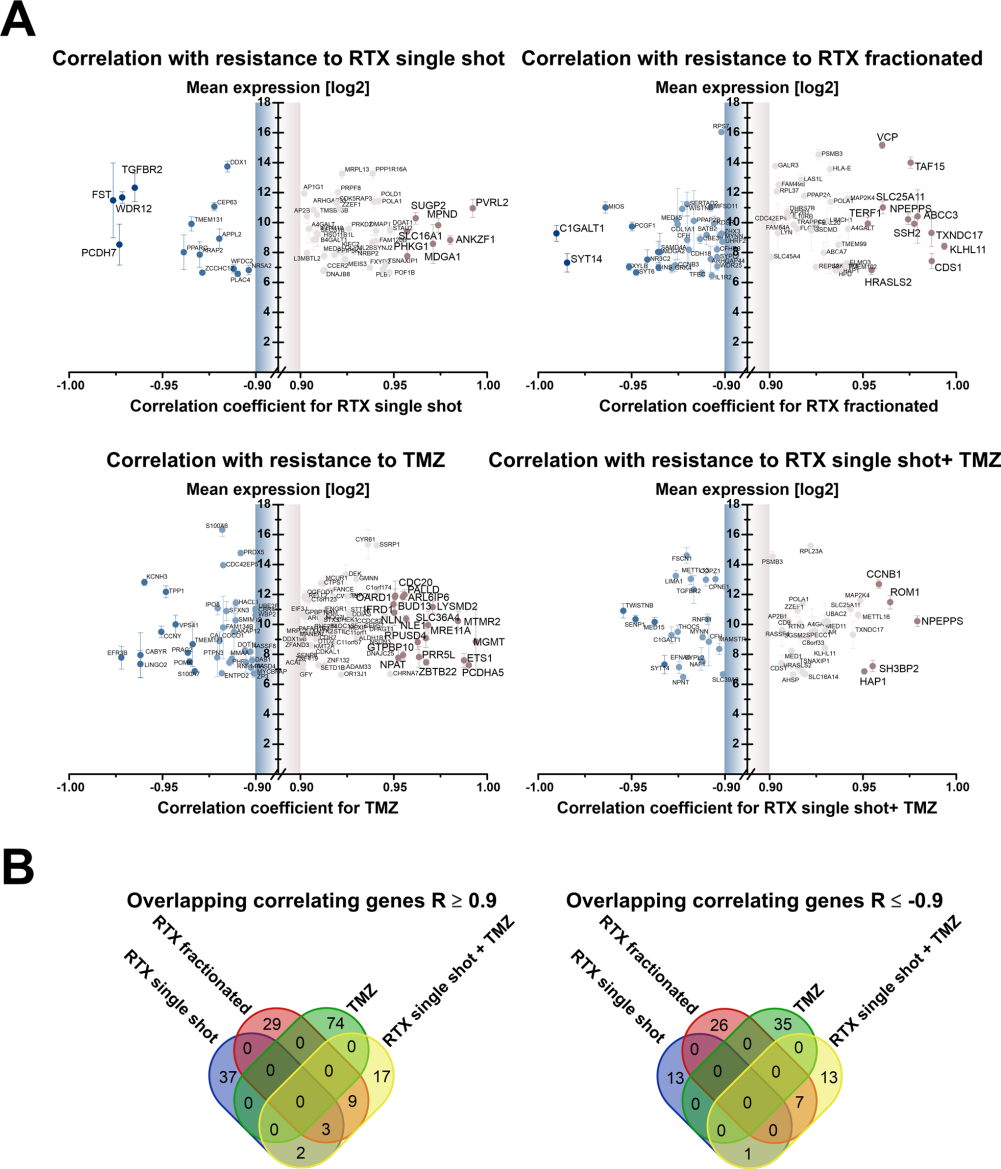
Integration of global mRNA expression data with scores of inherent therapy resistance in human glioblastoma cell lines. **a** Graphical representation of genes whose log2 mRNA expression levels show strong positive or negative correlation (|R|≥ 0.9) with inherent resistance to single-shot RTX, fractionated RTX, TMZ, and single-shot RTX + TMZ in human glioblastoma cell lines. Only genes with an average log2 expression value of 6 or higher compared to astrocytes are shown. **b** Intersect analysis of genes whose log2 mRNA expression shows significant positive or negative correlation (|R|≥ 0.9) with inherent resistance to single-shot RTX (blue), fractionated RTX (red), TMZ (green), and single-shot RTX + TMZ (yellow)

So far, our study has revealed that the annotation of defined molecular subtypes to established glioblastoma cell lines is challenging and yields different results depending on the molecular level and the key features employed. Furthermore, our data show that no direct association between the degree of therapy resistance and major transcriptomic differences or molecular subtypes can be drawn. Cell lines assigned to the highly treatment-refractory mesenchymal subtype (U138, U87) showed modest (U87) or even high (U138) sensitivity towards therapy, while the cell line U251 representing the classical subtype showed highest resistance (Fig. 2e). We therefore subjected the therapy resistance data and the transcriptomic profiling data of our glioblastoma cell line panel to correlation analyses with different workflows ([7], and Fig. 3). Firstly, utilizing whole transcriptome data, remaining on the single gene level, and setting a cutoff of |R|≥ 0.9 for the correlation of therapy resistance scores and log2 microarray expression values disclosed a limited set of genes whose expression levels were associated with resistance against IR and/or TMZ treatment. Most strikingly, the highest positive correlation for TMZ treatment (R > 0.99) was seen for O^6^-methylguanine-DNA-methyltransferase (MGMT), not only confirming the literature [48, 49], but also providing a very strong proof-of-concept for the feasibility of our methodological approach.

This strategy allowed us to identify 14 genes that exhibited significant positive correlation with inherent resistance to at least two of the four tested treatments ([7], and Fig. 3a, b), and 8 genes showing negative correlation (Fig. 3b and Additional file 1: Table S4). Three of the positively correlating genes were even linked to resistance towards three of the four treatments, including single-shot IR, fractionated IR, and single-shot IR plus TMZ (Fig. 3a, b). These genes encoded for alpha-1,4-galactosyltransferase (A4GALT), DNA polymerase alpha 1 (POLA1), a replication-associated DNA polymerase for which specific inhibitors are currently developed [50], and adaptor related protein complex 2 subunit beta 1 (AP2B1).

**Fig. 4:**
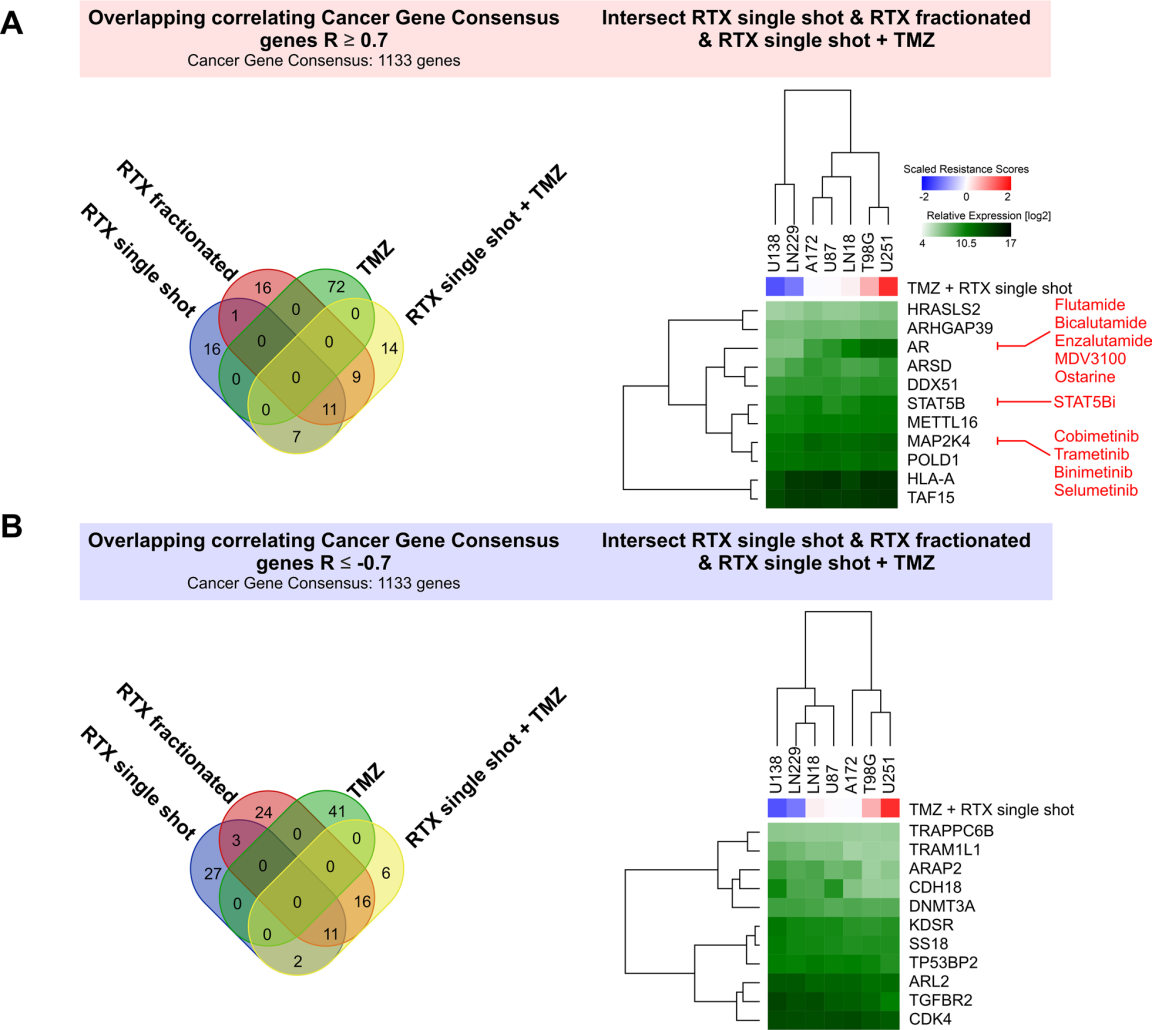
Integration of mRNA expression data of cancer gene consensus (CGC) genes with inherent therapy resistance in glioblastoma cell lines. **a** Intersect analysis of CGC genes whose log2 mRNA expression levels show significant positive (R ≥ 0.7) correlation with inherent resistance to single-shot RTX (blue), fractionated RTX (red), TMZ (green), and single-shot RTX + TMZ (yellow, left). Hierarchical clustering of relative log2 mRNA expression levels of 11 intersect genes correlating with resistance to single-shot RTX, fractionated RTX, and single-shot RTX + TMZ (right). Scaled scores of resistance to single-shot RTX + TMZ are depicted, and drugs antagonizing corresponding gene products are indicated in red. **b** Overlap analysis of CGC genes whose log2 mRNA expression shows significant negative (R ≤ -0.7) correlation with inherent resistance to single-shot RTX, fractionated RTX, TMZ and single-shot RTX + TMZ, and hierarchical clustering of relative log2 mRNA expression levels of 11 intersect genes

In order to focus on cancer-related genes, we next took advantage of the Cancer Gene Consensus (CGC) gene collection, which encompasses 1133 genes with documented functions in development and progression of cancer [25]. Using this compilation of reduced complexity compared to the whole transcriptome microarray and lowering the cut-off to |R|≥ 0.7 yielded a total of 27 cancer-related genes with positive, and 29 genes with negative correlation with resistance scores for at least two types of treatment (Fig. 4a, b). For more detailed analyses, we concentrated on genes, whose expression levels correlated with resistance against three types of treatment, resulting in 22 genes in total, 11 with positive correlation, and 11 with negative correlation (Fig. 4a, b). We then performed a search for drugs targeting the respective gene products with positive correlation with inherent therapy resistance (Fig. 4a and Additional file 1: Table S5). The most interesting target with the strongest expression dynamics across the cell line panel as identified by this approach was the androgen receptor (AR) which has recently been reported to play an important role in prognosis and therapy resistance of glioblastoma [51–57]. Since AR also has roles in other cancer entities, mostly in prostate and in breast cancer [58, 59], multiple inhibitors and antagonists of AR have been developed and trialed [60], yielding successful therapeutic targeting of the AR in these cancer entities [61, 62]. Another candidate for which targeted drugs with clinical approval in other cancer entities are readily available was the mitogen-activated protein kinase kinase 4 (MAP2K4) [63]. Interestingly, in case of glioblastoma a link between AR and MAPK signaling with regard to therapy resistance has recently been described [64]. Finally, our correlation analyses identified STAT5B as a druggable target [65, 66] whose expression was associated with therapy resistance, yet with rather low expression dynamics across the glioblastoma cell line panel.

### Gene set enrichment analyses (GSEAs) identify several signaling circuits as potential contributors to inherent therapy resistance in glioblastoma cells

**Fig. 5:**
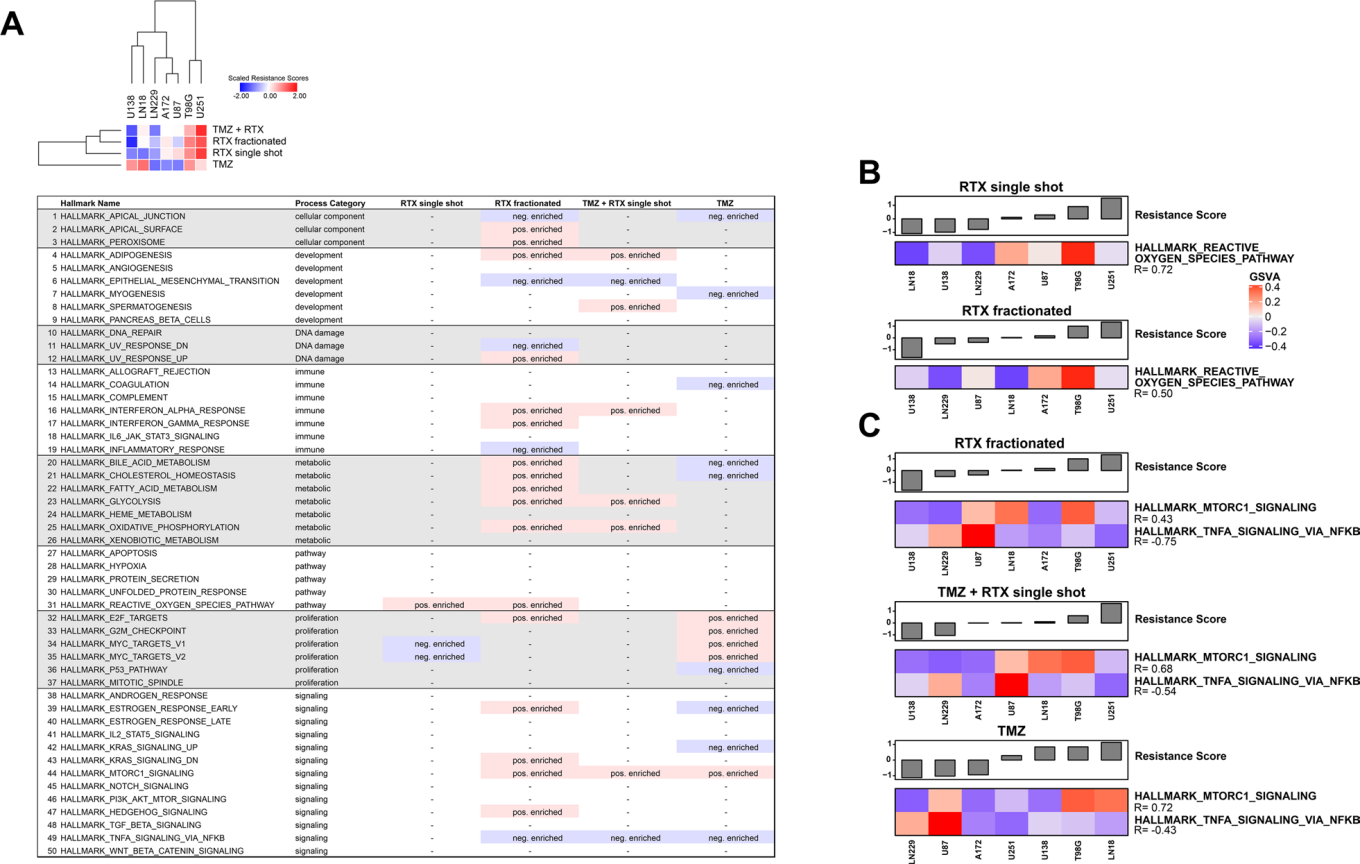
Gene set enrichment analysis (GSEA) on the basis of genes correlating with therapy resistance identifies pathways of potential contribution to therapy resistance in human glioblastoma cell lines. **a** Tabular presentation of pre-ranked gene set enrichment analysis (GSEA) results on the basis of obtained correlation coefficients (therapy resistance scores vs. gene expression data), and the MSigDB hallmarks collection [9] (FDR q-value cut-off ≤ 0.1). Positively enriched hallmark gene sets are depicted in pink, and negatively enriched hallmark gene sets are depicted in blue. **b, c** Correlation analysis of gene set variation indices as determined by gene set variation analysis (GSVA), and therapy resistance scores to fractionated RTX, single-shot RTX + TMZ, and sole TMZ

Stepping from the single gene to the gene set level, we performed pre-ranked gene set enrichment analyses (GSEAs) on the bases of the obtained correlation coefficients (Figs. 3 and 4) and the MSigDB Hallmarks collection which contains ground-truth derived gene sets reflecting the regulation of common biological processes [9]. With an FDR q-value cut-off of ≤ 0.1, a total of 21 gene sets with positive enrichment were found, and 14 gene sets with negative enrichment (Fig. 5a). Gene sets that showed positive/negative enrichment for resistance against at least two of the four treatments included process categories of the core metabolism, such as GLYCOLYSIS and OXIDATIVE_PHOSPHORYLATION, development (ADIPOGENESIS), and immune mechanisms (INTERFERON_ALPHA_RESPONSE), thus according with previous reports [67–73]. Hallmark gene sets found to be negatively enriched with resistance against at least two of the four treatments included EPITHELIAL_MESENCHYMAL_TRANSITION [74, 75] and APICAL_JUNCTION [76] (Fig. 5a). For resistance against treatments comprising IR, the hallmark gene set REACTIVE_OXYGEN_SPECIES_PATHWAY was positively enriched, and the maximal intersect for enrichment with resistance against three treatments was observed for MTORC1_SIGNALING (positive), and TNFA_SIGNALING_VIA_NFKB (negative). Inverting this workflow by first reducing the dimensionality of the expression data by single-sample gene set variation analysis (GSVA), followed by correlation analyses with the obtained GSVA scores and therapy resistance scores basically confirmed these results (Fig. 5b, c).

### Leading edge analyses (LEAs) of GSEA-derived gene sets identify druggable candidates and their functional interaction networks

**Fig. 6:**
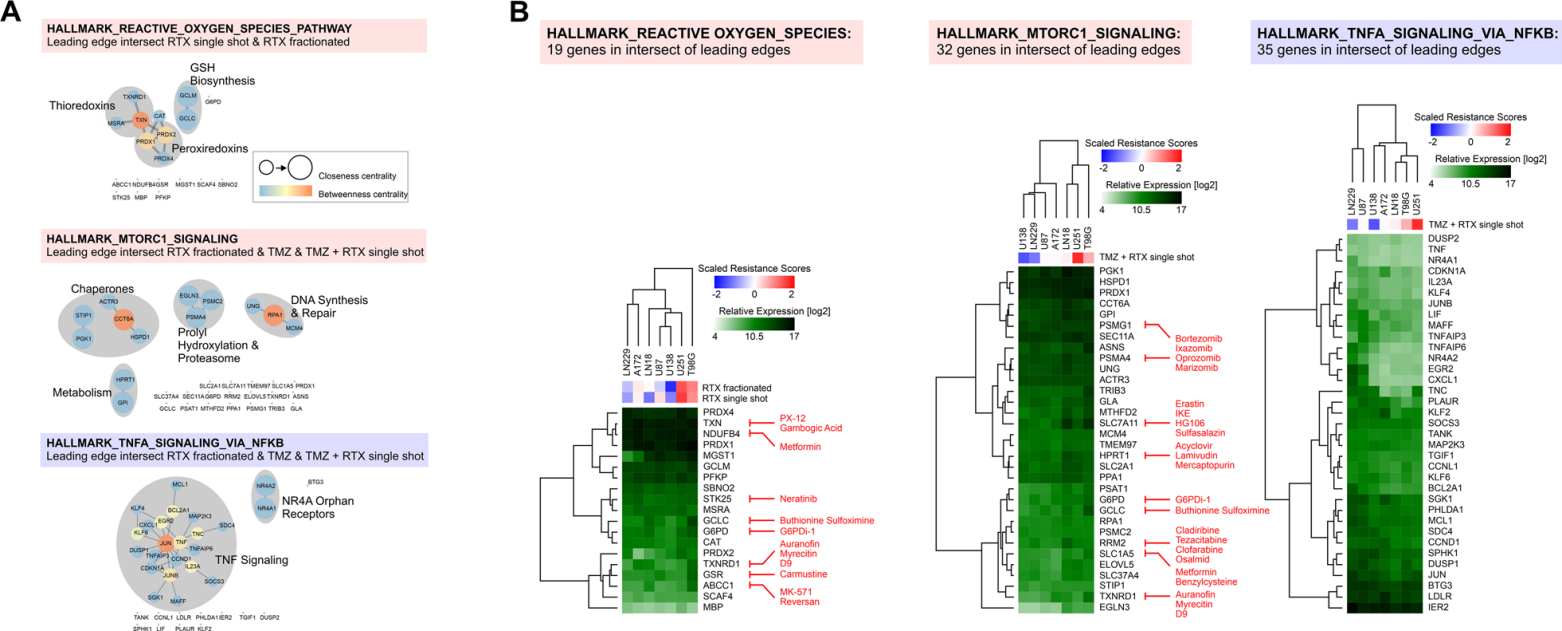
Leading edge analyses (LEAs) via functional interaction networks and hierarchical clustering. **a** Functional interaction networks of the leading edge intersect genes in hallmark gene sets REACTIVE_OXYGEN_SPECIES_PATHWAY, MTORC1_SIGNALING, and TNFA_SIGNALING_VIA_NFKB as identified by GSEA in Fig. 5A. **b** Hierarchical clustering of relative log2 mRNA expression levels of the respective leading edge intersect genes. Scaled scores of inherent therapy resistances are shown by unsupervised hierarchical clustering, and drugs antagonizing the respective gene products are indicated in red

Among the gene sets we identified to be positively or negatively enriched in therapy-resistant glioblastoma cell lines (Fig. 5a, b), we decided to concentrate on REACTIVE_OXYGEN_SPECIES_PATHWAY, MTORC1_SIGNALING and TNFA_SIGNALING_VIA_NFKB [10, 11, 77]. Leading edge analyses (LEAs) were performed by constructing functional interaction networks in Cytoscape. For REACTIVE_OXYGEN_SPECIES_PATHWAY, the leading edge genes comprised a circuit of thioredoxin/peroxiredoxin metabolism and glutathione (GSH) synthesis [78–80] (Fig. 6a and Additional file 1: Table S6) which in view of available drugs also appeared the most interesting vulnerabilities for sensitization in combined modality treatment approaches (Fig. 6b and Additional file 1: Table S6). Corresponding analyses for MTORC1_SIGNALING revealed druggable subnetworks involved in chaperoning, prolyl hydroxylation, proteasomal function, and DNA synthesis and repair, plus individual genes overlapping with thioredoxin/peroxiredoxin metabolism and GSH synthesis as disclosed in the leading edge of REACTIVE_OXYGEN_SPECIES_PATHWAY and involved in ferroptosis and autophagy regulation [81, 82] (Fig. 6a, b and Additional file 1: Table S6). Finally, the leading edge of negatively enriched TNFA_SIGNALING_VIA_NFKB was mainly composed of candidates directly involved in TNF/NF-κB signaling [83, 84] (Fig. 6a, b and Additional file 1: Table S6).

### Expression of candidate genes for targeted sensitization of glioblastoma as identified by GSEA and LEA is not driven by corresponding CNAs and only marginally anti-correlates with respective promotor methylation

**Fig. 7:**
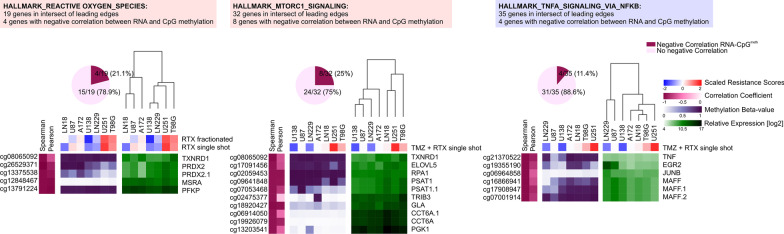
CpG methylation status of leading edge intersect genes shows poor correlation between DNA methylation and therapy resistance. Correlation analyses of DNA methylation beta values (shown in purple) and relative log2 mRNA expression levels (shown in green) of leading edge intersect genes of the REACTIVE_OXYGEN_SPECIES_PATHWAY, MTORC1_SIGNALING, and TNFA_SIGNALING_VIA_NFKB hallmark gene sets. Coefficients of significant negative correlation are depicted by heat map clustering (shown in dark pink), and scaled resistance scores to single-shot and fractionated RTX (REACTIVE_OXYGEN_SPECIES_PATHWAY), or single-shot RTX + TMZ (MTORC1_SIGNALING, and TNFA_SIGNALING_VIA_NFKB) are shown by unsupervised hierarchical clustering

In order to examine if expression of the leading edge genes identified by our correlation/GSEA workflow is reflected by CNAs on the DNA level or by CpG methylation, we integrated the corresponding data sets. Unexpectedly, no significant association between the transcriptome and the CNA level was observed (not shown). Furthermore, significant negative correlation with promotor methylation was only observed for a minor subset of genes (Fig. 7) suggesting that other mechanisms dominate gene expression in this context and that integrative approaches such as the one described in the present study require mRNA expression data, rather than CNA or DNA methylation data.

In summary, our data show that workflows correlating mRNA expression data with PCA-derived scores of inherent therapy resistance, followed by GSEA and LEA can reveal therapy resistance markers and potential vulnerabilities for pharmacological sensitization—both previously reported ones and hitherto unknown candidates, and thereby can open new perspectives for mechanism-based, combined modality treatment of glioblastoma and probably also of other cancer entities.

## Discussion

Inherent therapy resistance is a major challenge in the treatment of various malignancies. Glioblastoma is particularly well-known for its high degree of treatment resistance, accounting for its dismal prognosis [4]. The identification of key regulators that orchestrate this resistance is therefore inevitable in order to disclose novel perspectives of targeted combined modality therapy and to improve treatment outcome [29]. We recently showed that integrating scores of inherent therapy resistance as extracted from clonogenic survival data with mRNA expression data of the DNA damage response is a suitable approach to identify new candidates for targeted sensitization of glioblastoma [7, 8]. Here, we expanded this workflow to global mRNA expression data and additional molecular levels, including DNA methylome and chromosomal CNAs—all collected under treatment-naive conditions which most closely resemble the clinical situation of tissue sampling in form of tumor biopsies and resections. In accordance with our preceding study, the identification of O^6^-methylguanine-DNA-methyltransferase (MGMT) as the best correlating candidate among all genes with positive correlation of mRNA expression with resistance to TMZ provided a proof-of-concept for the feasibility of our approach. Furthermore, on the single gene level, we identified the androgen receptor (AR) as a crucial positive correlator with inherent resistance to three of the four types of treatment—a candidate which is already successfully therapeutically addressed in several cancer entities, such as prostate cancer [58, 61, 85], and thus is eligible to rapid evaluation in glioblastoma [52]. Another gene whose expression level correlated positively with resistance to three types of glioblastoma treatment was mitogen-activated protein kinase kinase 4 (MAP2K4) [63]. Various inhibitors of the MAP kinase family have been developed and several of them, including trametinib and cobimetinib, are readily approved for clinical use in other cancer entities [86]. However, it is known that these compounds commonly do not pass the blood brain barrier (BBB) which for instance undermines efficient treatment of brain metastases originating from MAP kinase-driven melanoma [87, 88]. Therefore, new BBB-passing MAP kinase inhibitors such as E6201 are currently in development [89–92]. Our data suggest that these inhibitors may be interesting candidates for targeted sensitization of glioblastoma to IR and/or TMZ.

In order to identify gene sets, networks, and signaling circuits rather than single genes, we made use of pre-ranked gene set analyses (GSEAs) on the basis of the correlation coefficients (gene expression vs. therapy resistance) followed by leading edge analyses (LEAs). This indeed yielded several candidates whose pharmacological targeting appears interesting in the context of targeted radio- and/or chemosensitization of glioblastoma and—more importantly—for which refined drugs are readily available. Among these were the MSigDB hallmark gene sets REACTIVE_OXYGEN_SPECIES_PATHWAY and MTORC1_SIGNALING, both of which comprise candidates that can be well-targeted by various available drugs [93, 94]. Mechanistically, the identified regulatory circuits have major implications for death and/or survival pathways, such as ferroptosis and autophagy [81, 95], and crucial pro-survival players would represent interesting targets in order to break glioblastoma cell death evasion and resistance against IR [96–98] or TMZ [99], respectively.

When tracing the mRNA expression data back to the chromosomal CNA status, we did not observe relevant associations. Furthermore, CpG methylation status did only in part reflect the obtained mRNA expression data. Accordingly, other mechanisms, including posttranscriptional regulation of mRNA expression by micro-RNAs (miRNAs) [100], may be involved in glioblastoma gene regulation accounting for the observed therapy resistance-associated mRNA expression patterns, and this would also fit with several recent reports identifying miRNA signatures as outcome prognosticators of glioblastoma [101–104]. It is in accordance with previous reports which found only marginal associations between the methylation status of MGMT promotor and mRNA or protein expression levels in glioblastoma patients [49, 105]. Hence, it is feasible to assume that our integrative approach requires mRNA expression data, rather than DNA methylation or CNA data to obtain robust results. In how far (phospho-)proteomic data would further improve the study outcome remains to be investigated [106].

Similar systematic in vitro analyses of therapy resistance on the basis of clonogenic survival data and multi-level molecular data are rare [107, 108], not only in glioblastoma [109–111]. Most studies of similar purpose integrate molecular data from patient material provided by publicly available databases such as TCGA with clinical data in order to identify genes with association to therapy resistance [22, 112–114]. However, the molecular data obtained from such cohorts are often of high complexity given that biopsied/resected patient material usually comprises complex tumor tissue, including tumor stroma and normal tissue cells rather than only tumor cells [115, 116]. Furthermore, information on treatment courses and clinical endpoints commonly is scarce and/or incomplete, thus further hampering the interpretability of these data. Our results do not only provide proof-of-concept for the feasibility of the chosen integrative in vitro approach. The data sets generated in the present study comprising functional (clonogenic survival data) and multi-level molecular data (mRNA transcriptome, DNA methylome, chromosomal CNA, and SKY FISH) of very commonly used glioblastoma cell lines (in an unperturbed, untreated stage) also represent a valuable toolbox which can be readily interrogated by other researchers in the field of glioblastoma therapy resistance. Nevertheless, since dynamic changes on the analyzed molecular levels (particularly on the transcriptome and on the methylome level) are to be expected in response to therapy and may well be causative for the emergence of acquired therapy resistance [30, 117–119], further research is certainly needed. Finally, the integrative nature of the described workflow can be adapted to other disease models, such as 3D cell culture and organoids [120], since this may impact both, global mRNA expression and therapy resistance [121–123].

## Supplementary Information

**Additional file 1.** Supplementary tables.

**Additional file 2.** Compilation of all molecular data collected in this study.

## Data Availability

The gene expression data and the array CGH data presented in this study are publicly available at Gene Expression Omnibus (GEO) under the super set accession number: GSE119637. For the review process, reviewers can access the data under the link https://www.ncbi.nih.gov/geo/query/acc.cgi?acc=GSE119637 using the token ‘sfqnocumrvofrmv’.

## Abbreviations

A4GALT: Lactosylceramide 4-alpha-galactosyltransferase
ABC: ATP binding cassette
ABCC1: ATP binding cassette subfamily C member 1
aCGH: Array comparative genomic hybridization
ACTR3: Actin related protein 3
AKT: V-Akt murine thymoma viral oncogene
AKT1: V-Akt murine thymoma viral oncogene 1
ANSI: American National Standards Institute
AP2: Adaptor related protein complex 2
AP2B1: Adaptor related protein complex 2 subunit beta 1
AR: Androgen receptor
ARAP2: ArfGAP with RhoGAP domain, ankyrin repeat and PH Domain 2
ARHGAP39: Rho GTPase activating protein 39
ARL2: ADP ribosylation factor like GTPase 2
ARSD: Arylsulfatase D
ASCT2: Alanine serine cysteine transporter 2
ASNS: Asparagine synthetase
ATCC: American Type Culture Collection
BBB: Blood brain barrier
BCL2A1: BCL2 related protein A1
BCR: BTB-Cul3-Rbx1
BTB: BR–C, Ttk and bab
BTG3: BTG anti-proliferation factor 3
CASP1: Caspase-1
CAT: Catalase
C1GALT1: Core 1 glycoprotein-N-acetyl-galactosamine-3-beta-galactosyltransferase 1
CCND1: Cyclin D1
CCNL1: Cyclin L1
CCT6A: Chaperonin containing TCP1 subunit 6A
CDH18: Cadherin 18
CDK4: Cyclin dependent kinase 4
CDKN1A: Cyclin dependent kinase inhibitor 1A
CDS1: Phosphatidate cytidylyltransferase 1
CFH: Complement factor H
CGC: Cancer gene consensus
CHI3L1: Chitinase-3-like protein 1
CIMP: CpG island methylator phenotype
CIN: Chromosomal instability
CNA: Copy number alteration
COSMIC: Catalogue of somatic mutations in cancer
CSF1: Colony stimulating factor 1
CUL3: Cullin 3
CXCL1: C-X-C motif chemokine ligand 1
CXCR4: C-X-C motif chemokine receptor 4
D-MEM: Dulbecco’s modified eagle medium
DAPI: 4′,6-Diamidino-2-phenylindole
DDR: DNA damage response
DDX51: DEAD-box helicase 51
DNMT3A: DNA methyltransferase 3 alpha
DUSP1: Dual specificity phosphatase 1
DUSP2: Dual specificity phosphatase 2
EGFR: Epidermal growth factor receptor
EGLN3: Egl-9 family hypoxia inducible factor 3
EGFRvIII: Epidermal growth factor receptor variant III
EGR2: Early growth response 2
ELOVL5: Fatty acid elongase 5
EMT: Epithelial-mesenchymal transition
ERK: Extracellular signal-regulated kinase
FCS: Fetal calf serum
FDR: False recovery rate
FIR: Fractionated IR
FOXP3: Forkhead box protein 3
G6PD: Glucose-6-phosphate dehydrogenase
GCKIII: Germinal centre kinase III
GCLC: Glutamate-cysteine ligase catalytic subunit
GCLM: Glutamate-cysteine ligase modifier subunit
GEO: Gene expression omnibus
GLA: Galactosidase alpha
GPI: Glucose-6-phosphate isomerase
GSC: Glioma stem cell
GSEA: Gene set enrichment analysis
GSH: Glutathione
GSR: Glutathione-disulfide reductase
GSSG: Gluthathione disulfide
GSVA: Gene set variation analysis
HLA-A: Major histocompatibility complex, class I, A
HPRT1: Hypoxanthine phosphoribosyltransferase 1
HRASLS2: HRAS-like suppressor 2
HSPD1: Heat shock protein family D member 1
HAP1: Huntingtin-associated protein 1
HTT: Huntingtin
IDO1: Indoleamine 2,3-dioxygenase 1
IER2: Immediate early response gene 2
IL2: Interleukin 2
IL2RA: Interleukin-2 receptor alpha
IL4: Interleukin 4
IL23A: Interleukin 23 subunit alpha
IR: Ionizing radiation
ISCN: International system for cytogenetic nomenclature
JAK: Janus kinase
JNK: C-Jun N-terminal kinase
JUN: Jun proto-oncogene
JUNB: Jun B proto-oncogene
KDSR: 3-Ketodihydrosphingosine reductase
KEAP1: Kelch like ECH associated protein 1
KLF2: Kruppel like factor 2
KLF4: Kruppel like factor 4
KLF6: Kruppel like factor 6
KLHL11: Kelch-like family member 11
KLK2: Kallikrein related peptidase 2
KLK3: Kallikrein related peptidase 3
LDLR: Low density lipoprotein receptor
LEA: Leading edge analysis
LGG: Low-grade glioma
LIF: Interleukin 6 family cytokine
LKB1: Liver kinase B1
MAFF: MAF BZIP transcription factor F
MAP: Mitogen-activated protein kinase
MAP2K3: Mitogen-activated protein kinase kinase 3
MAP2K4: Mitogen-activated protein kinase kinase 4
MAPK: Mitogen-activated protein kinase
MBP: Myelin basic protein
MC: Mediator complex
MCL1: Induced myeloid leukemia cell differentiation protein Mcl-1
MCM4: Minichromosome maintenance complex component 4
MCM15: Mediator complex subunit 15
METTL16: Methyltransferase 16
MGMT: O^6^-methylguanine-DNA-methyltransferase
MGST1: Microsomal glutathione S-transferase 1
miRNA: MicroRNA
MSRA: Methionine sulfoxide reductase A
MTHFD2: Methylene-tetrahydrofolate dehydrogenase 2
mTORC1: Mammalian target of rapamycin complex 1
MYNN: Myoneurin
NDUFB4: NADH-ubiquinone oxidoreductase subunit B4
NES: Nestin
NF-κB: Nuclear factor κB
NF1: Neurofibromin
NOTCH3: Notch receptor 3
NPEPPS: Puromycin-sensitive aminopeptidase M1
NR4A1: Nuclear receptor subfamily 4 group A member 1
NR4A2: Nuclear receptor subfamily 4 group A member 2
NRF2: NF-2-related factor 2
NSCLC: Non-small cell lung cancer
PBS: Phosphate-buffered saline
PCA: Principal component analysis
PDGFR: Platelet-derived growth factor receptor
PDGFRA: Platelet-derived growth factor receptor alpha
PFKP: Phosphofructokinase, platelet
PGK1: Phosphoglycerate kinase 1
PHLDA1: Pleckstrin homology like domain family A member 1
PI3K: Phosphatidylinositol 3-kinase
PLAAT2: Phospholipase A and acyltransferase 2
PLAUR: Plasminogen activator, urokinase receptor
POL1: RNA polymerase 1
POLA1: DNA polymerase alpha 1, catalytic subunit
POLD1: DNA polymerase delta 1, catalytic subunit
POLR1F: RNA polymerase 1 subunit F
PPA1: Inorganic pyrophosphatase 1
PRDX1: Peroxiredoxin 1
PRDX2: Peroxiredoxin 2
PRDX4: Peroxiredoxin 4
PRL: Prolactin
PSAT1: Phosphoserine aminotransferase 1
PSMA4: Proteasomal 20S subunit alpha 4
PSMB3: Proteasomal subunit beta 3
PSMC2: Proteasome 26S subunit, ATPase 2
PSMG1: Proteasome assembly chaperone 1
PSMG2: Proteasome assembly chaperone 2
PSMG4: Proteasome assembly chaperone 4
RAS: Rat sarcoma
RBX1: Ring box 1
RELB: Transcription factor RelB
RING: Really interesting new gene
RNAseq: RNA sequencing
ROS: Reactive oxygen species
RPA1: Replication protein A1
rRNA: Ribosomal RNA
RRM2: Ribonucleotide reductase regulatory subunit M2
RTX: Radiotherapy
SAP: Stress-activated protein
SBNO2: Strawberry Notch homolog 2
SCAF4: SR-related CTD associated factor 4
SDC4: Syndecan 4
SEC11A: SEC11 homolog A
SGK1: Serum/glucocorticoid regulated kinase 1
SKY FISH: Spectral karyotyping fluorescent in situ hybridization
SLC1A5: Solute carrier family 1 member 5
SLC2A1: Solute carrier family 2 member 1
SLC7A11: Solute carrier family 7 member 11
SLC25A11: Solute carrier family 25 member 11
SLC37A4: Solute carrier family 37 member 4
SMO: Smoothened
SNP: Single nucleotide polymorphism
SOCS3: Suppressor of cytokine signaling 3
SOD1: Superoxide dismutase 1
SOX: Sry-type HMG box
SOX2: Sry-type HMG box 2
SOX10: Sry-type HMG box 10
SPHK1: Sphingosine kinase 1
SS18: SS18 subunit of BAF chromatin remodeling complex
ssGSEA: Single-sample GSEA
SSIR: Single-shot IR
STAT: Signal transducer and activator of transcription
STAT5B: Signal transducer and activator of transcription 5B
STIP1: Stress induced phosphoprotein 1
STK25: Serine/threonine kinase 25
STR: Short tandem repeat
SYPL1: Synaptophysin-like 1
SYPL2: Synaptophysin-like 2
SYT14: Synaptotagmin-14
TAF15: TATA-box binding protein associated factor 15
TANK: TRAF family member associated NF-κB activator
TGFBR1: Transforming growth factor beta receptor 1
TGFBR2: Transforming growth factor beta receptor 2
TGIF1: TGFβ induced factor homeobox 1
TMEM97: Transmembrane protein 97
TMZ: Temozolomide
TNC: Tenascin C
TNF: Tumor necrosis factor
TNFA: Tumor necrosis factor alpha
TNFAIP3: Tumor necrosis factor alpha induced protein 3
TNFAIP6: Tumor necrosis factor alpha induced protein 6
TNFRSF1A: Tumor necrosis factor receptor super family member 1A
TP53BP2: Tumor protein p53 binding protein 2
TRADD: TNFRSF1A associated via death domain
TRAM1L1: Translocation associated membrane protein 1 like 1
TRAPPC6B: Trafficking protein particle complex, subunit 6B
TRIB3: Tribbles pseudokinase 3
TRX: Thioredoxin
TSNAXIP1: Translin-associated X-interacting protein 1
TWISTNB: TWIST neighbor
TXN: Thioredoxin
TXNDC17: Thioredoxin domain containing protein 17
TXNRD1: Thioredoxin reductase 1
UNG: Uracil DNA glycosylase
WNT: Wingless and Int-1
ZDHHC17: Zinc finger DHHC-type palmitoyltransferase 17
ZZEF1: ZZ-type zinc finger and EF-hand domain-containing protein 1
