# Supplementary material for: Systematic in vitro analysis of therapy resistance in glioblastoma cell lines by integration of clonogenic survival data with multi-level molecular data
Source: Radiat Oncol. 2023 Mar 11;18:51. doi: 10.1186/s13014-023-02241-4 (PMC10007763; doi:10.1186/s13014-023-02241-4)
Supplement: Supplementary file 1 — Additional file 1. Supplementary tables. [file 13014_2023_2241_MOESM1_ESM.pdf]

Supplementary Table 1: Principal component analysis (PCA)-derived scores of inherent resistance to single-shot radiotherapy (RTX), fractionated RTX, temozolomide (TMZ), and the combination thereof in human glioblastoma cell lines A172, LN18, LN229, T98G, U87, U138, and U251

	**A172**	**LN18**	**T98G**	**U138**	**U87**	**LN229**	**U251**
**RRID**	CVCL-0131	CVCL-0392	CVCL_0556	CVCL_0020	CVCL_0022	CVCL-0393	CVCL-0021
Single-shot RTX	0.11	-1.08	0.91	-0.98	0.29	-0.77	1.53
Fractionated RTX	0.18	0.03	0.99	-1.67	-0.38	-0.50	1.35
TMZ	-0.94	1.13	0.84	0.84	-1.03	-1.13	0.29
Single-shot RTX/TMZ	0.00	0.11	0.61	-1.34	0.02	-1.04	1.65

Supplementary Table 2: Overview of composite karyotypes in human glioblastoma cell lines A172, LN18, LN229, T98G, U87, U138, and U251

Cell line	Composite clonal karyotype	Subclones
**A172**	83,XXYY,der(1)t(1;14)(p13;?),der(1)t(1;18)(p22;?),+der(1)del(1)(p11)t(1;14)(q21;?),+del(1)(p22;q11),+der(2)t(2;5)(q31;?)x2,+der(3)(3;9)(q25;?),+5,+der(5)del(5)(q15)t(2;5)(?;p13)x2,+6,+7,+der(7)t(6;7)(q22;?),del(8)(q23),+del(8)(q11),+del(8)(q13),der(9)t(9;16)(p11;?)x2,+10,+11,+der(11)t(1;11)(?;p11)x2,+12,+12,+13,+der(14)t(9;14)(?;q13)x2,+15,+16,+16,+16,+17,+der(17)t(17;18)(q11;?),der(18)t(1;18)(?;q21),+der(18)t(1;18)(?;q21),+19,+19,+20,+20,add(21)(q21),+22	Yes
**LN18**	61,XY,+t(X;10)(q?;q11),+t(X;9)(p?;p?),+3,+t(5;15)(p13;q?),+7,+7,t(6;10)(q11;q?),+t(5;10)(q11;q?),+t(6;10)(p11;p?),+t(10)(p10),t(11;14)(q?;?),+t(11,18)(q?;q11),+t(12;16)(q?;p11),+20,+t(16;20)(p11;q?)+add(22)(q?)	Yes
**LN229**	86,XXX,der(X)t(x;2)(q23;?),der(X)t(X;16)(p11;?),+der(X)t(X;19)(p21;?),+1,del(1)(p10),+der(1)t(1;18)(?;q11),+2,+2,+2,+3,+3,+der(4)t(4;6)(p14;?)x2,+der(4)t(4;9)(q11;?),+5,+der(5)t(5;12)(p11;?),+der(6)t(6;16)(p13;?)x2,+der(6)t(6;7)(?;?),+7,+7,+der(7)t(5;7)(?;p11)x2,+der(8)t(6;8)(?;q22),+9,+9,+der(9)t(8;9)(?;q10),+10,+der(11)(q14),+del(12)(q13),+der(14)t(14;18)(q24;?),+16,+16,+del(17)(q12),+18,+der(19)t(X;19)(?;q12),+20,+20,+20,+21,+21,+22,+22,+der(22)t(15;22)(?;p12)x2	Yes
**T98G**	131,XXXYYYY,trc(7;7;7)(q?;p?;p?)x2,+t(7;8)(q?;p?)x2,dic(10;10)(q?;q?)x1,+i(15)(q10)	Yes
**U87**	44,X,t(1)(1:13)(p22;p?)x2,der(6)t(6;7)(p21;p?),der(6)t(6;12)(q23;?),del(7)(q10),del(10)(q21),der(12)t(6;12)(?;q22),-13,-14,der(16)t(1;16)(?;q13),del(20)(p11),+der(20)t(1;14;20)(?;?;q12),t(10;22)(?;p12)	Yes
**U138**	61-62,XYY,+Y,+der(1)t(1;12)(p31;?),+3,der(4)t(4;17)(q32;?),ins(4)(4;14;4)(p15;?;p13)x2,+7,+der(8)t(8;17)(p21;?),+der(11)t(11;19)(p13;?),der(12)t(1;12)(?;q14),+13,-14,+15,+18,+der(18)t(6;18)(?;q12),+del(19)(p12),+20,+20,+22,+der(22)t(5;22)(?;q11)	No
**U251**	66,XXY,+1,+2,+3,der(4)t(4;16;4;20)(q12;?;q?;q?),der(4)t(4;16;4;16)(q12;?;q?;?),+5,+7,+7,+iso(8)(q10),+9,+der(11)t(10;11;15)(q10;q?;q?),+der(11)t(6;10;11)(q?;q?;q?),+15,+17,+17,+del(18)(q12),+19,+20,+21	No

The second column describes the predominant clonal karyotype including all chromosomal aberrations. The third column shows whether subclones are present in the cell lines.

Supplementary Table 3: Overview of molecular glioblastoma subtype-specific chromosomal amplifications and deletions, and subtype-specific expression of relevant driver genes

Subtype	Feature	A172	LN18	LN229	T98G	U87	U138	U251
Classical	EGFR (7p11.2) amplification	1	1	1	1	0	0	1
	CDKN2A (9p21.3) deletion	1	1	1	1	1	1	1
	NOTCH3 expression	1	0	0	0	1	0	0
	NES expression	1	0	1	0	1	0	1
	SMO expression	1	0	1	1	0	0	1
Score classical		1.0	0.4	0.8	0.6	0.6	0.2	0.8
Mesenchymal	NF1 (17q11.2) deletion	0	0	1	0	0	1	0
	CHI3L1 expression	0	0	0	0	1	0	0
	TRADD, RELB, TNFRSF1A expression	1	1	1	1	1	1	1
	CASP1 espression	0	1	0	1	1	1	1
	TLR4 expression	0	0	1	0	0	0	1
Score mesenchymal		0.2	0.4	0.6	0.4	0.6	0.6	0.6
Proneural	PDGFRA (4q12) amplification	0	0	0	0	0	0	1
	NKX2-2 expression	0	0	1	0	0	0	0
	OLIG2 expression	0	0	1	0	0	0	0
	SOX2 expression	0	0	1	0	0	0	0
	ERBB3 expression	0	0	1	0	0	0	0
Score proneural		0	0	0.8	0	0	0	0.2
Classification key feature-based		C	C/M	C/P	C	C/M	M	C

Supplementary Table 4: Genes with overlapping positive (R ≥ 0.9, red) or negative (R ≤ -0.9, blue) correlation with inherent therapy resistance in human glioblastoma cell lines as retrieved by global mRNA expression microarray analysis

Gene ID	SSIR	FIR	COMBI	TMZ	General information	Connection to glioblastoma	Inhibitors	Clinical trials
A4GALT	> 0.9	> 0.9	> 0.9	-	Lactosylceramide-4-alpha-galactosyltransferase (A4GALT) catalyzes the transfer of galactose residues onto sphingolipids thereby contributing to the generation of glycosphingolipids [1, 2].	Connections between A4GALT and glioblastoma are not reported so far.<br><br>In lung cancer models, A4GALT was shown to affect tumor progression, metastasis formation, resistance to chemotherapy, and epthelial-mesenchymal transition (EMT) [3].	None	None
POLA1	> 0.9	> 0.9	> 0.9	-	DNA polymerase alpha 1 (POLA1) catalytic subunit, together with one regulatory and two primase subunits, forms the DNA polymerase alpha (POLA) complex. POLA1 is important for the initiation of DNA replication as it synthesizes RNA:DNA hybrids thereby initiating the synthesis of Okazaki fragments [4-7]. POLA1 also synthesizes cytosolic RNA:DNA hybrids thereby modulating the type I interferon immune response [8].	Connections between POLA1 and glioblastoma are not reported so far. However, MIR075, an unpublished POLA1 inhibitor, reduced the growth of glioblastomas generated by intracranial implantation of U87 cells into mice (doi.org/10.1158/1538-7445.AM2018-48489).<br><br>POLA1 is of prognostic value in non-small cell lung cancer (NSCLC), and colorectal cancer [9-12].	CD437 [13]<br>ST1926 [14, 15]<br>MIR002 [16]<br>GEM144 [16]<br>(co-inhibits HDAC11)<br>MIR020,<br>MIR072,<br>MIR075<br>(all unpublished)	None
AP2B1	> 0.9	> 0.9	> 0.9	-	AP2 complex subunit Beta 1 (AP2 B1) is one of two components that form the Assembly Protein 2 (AP2) complex which links clathrin to its receptors on vesicles. Thus, AP2B1 is important for intracellular vesicle trafficking and endocytosis [17].	Connections between AP2B1 and glioblastoma are not reported so far, but have been suggested [18].<br><br>Aberrancies in expression of AP2B1 and expression of alternative splicing forms of AP2B1 were detected in different cancer entities including lung cancer and breast cancer [19, 20].	Barbadin [21]<br>(co-inhibits beta-Arrestin)	None
TSNAXIP1	> 0.9	-	> 0.9	-	Translin-Associated X-Interacting Protein 1 (TSNAXIP1) is a so far uncharacterized protein predicted to be involved in cell proliferation, cell differentiation, cell polarity, and spermatogenesis [22].	TSNAXIP1 was identified in a nomogram of alternative splicing forms that were of predictive value for low-grade gliomas [23].	None	None
ZZEF1	> 0.9	-	> 0.9	-	ZZ-type zinc finger and EF-hand domain-containing protein 1 (ZZEF1) specifically detects histone H3 at promoters thereby functioning co-activator of transcription [24].	ZZEF1 is deleted in a notable portion of pediatric gliomas [25].<br><br>Single-nucleotide polymorphisms (SNPs) and copy number alterations (CNAs) of ZZEF1 are associated with several types of cancer including pancreatic cancer, gastric cancer, breast cancer, and esophageal cancer [26].	None	None

MAP2K4	-	> 0.9	> 0.9	-	Dual specificity Mitogen-Activated Kinase Kinase 4 (MAP2K4/MEK4/ MKK4) is a part of the MAPK (RAF-MEK-ERK) and Stress-Activated Protein (SAP) Kinase/c-Jun N-terminal Kinase (JNK) signaling pathway network [27].	MAP2K4 exhibits tumor suppressing functions by decelerating tumor progression and metastasis formation [28-33]. MAP2K4 is frequently mutated in cancer, and mutations of MAP2K4 exhibit proto-oncogenic potential [34-40]. In addition, mutations of MAP2K4 frequently conincide with resistances to MAPK-targeting therapies [41].<br><br>MAP2K4, together with ZDHHC17 and JNK/p38, contributes to progression of glioblastoma by promoting the self-renewal of glioma stem cells (GSCs) [42]. This effect can be inhibited by Genistein, an isoflavone protein tyrosine kinase (PTK) inhibitor derived from soy bean [43] which disrupts the interaction between MAP2K4 and ZDHHC17 in glioblastoma cells [42]. Genistein synergizes with radiotherapy and temozolomide (TMZ) in terms of abrogating survival of glioblastoma cells *in vitro* [44, 45]. Finally, expression of miR-744, a MAP2K4-targeting miRNA interferes with migration and invasiveness of glioblastoma cells *in vitro* [46].	MEK4 inhibitor-1 [47]<br>MEK4 inhibitor-2 [47]<br>3-Arylimidazoles [48]<br>BSJ-04-122 [49] (co-inhibits MEK7)<br>HRX-0215 (unpublished)<br><br>Genistein/NPI031L [43] (PTK inhibitor)	Genistein was tested in multiple trials on patients suffering from different diseases including different malignancies, but not in glioblastoma so far [50].<br><br>Currently, Genistein is tested in combination with systemic chemotherapy in a randomized trial on pediatric patients either suffering from lymphomas or different kinds of solid tumors including pediatric gliomas (NCT02624388).
PLAAT2/ HRASLS 2	-	> 0.9	> 0.9	-	Phospholipase A and Acyltransferase 2 (PLAAT2/HRASLS2) exhibits two enzymatic activities, a phospholipase and an acyltransferase activity. PLAAT2/HRASLS2 catalyzes the hydrolysis of dipalmitoylated phosphatidylcholine into lysophosphatidylcholin and palmitic acid as well as the acylation of phosphatidylethanolamine [51, 52].	PLAAT2/HRASLS2 is differentially expressed in the stromal cells of glioblastoma tumors [53].<br><br>PLAAT2/HRASLS2 exhibits tumor suppressive functions, most likely by attenuating the oncogenic potential of RAS GTPase [51].	LEI110 [54] (pan-HRASLS inhibitor)<br>LEI301 [55] (pan-HRASLS inhibitor)	None
CDS1	-	> 0.9	> 0.9	-	Phosphatidate Cytidylyltransferase 1 (CDS1) catalyzes the conversion of phosphatidic acid into CDP-diacylglycerol. CDS1 is involved in syntheses of phosphatidylinositol, phosphatidylglycerol, and cardiolipin. Thus, CDS1 regulates the intracellular pool of the signal transduction-related second messenger phosphatidylinositol [56, 57].	Alterations in mRNA expression levels of CDS1 are associated with poor prognosis in glioblastoma and liver cancer [58, 59]. Functionally, CDS1 was shown to affect the lipid metabolism of glioblastoma cells upon hypoxic stress [60].	None	None
SLC25A1 1	-	> 0.9	> 0.9	-	The Solute Carrier family 25 member 11 (SLC25A11) catalyzes the electron-neutral transport of 2-oxoglutarate across mitochondrial membranes thereby allowing for the synthesis of malate and other dicarboxylic acids. SLC25A11 is involved in the generation of ATP and in maintenance of the redox equilibrium [61].	Connections between SLC25A11 and glioblastoma are not reported so far.<br><br>SLC25A11 is deregulated in several types of malignancy including NSCLC, liver cancer, and melanoma [62, 63]. Germline mutations of SLC25A11 are associated with increased susceptibilty for developing paragangliomas [64].	None	None
HAP1	-	> 0.9	> 0.9	-	Huntingtin-Associated Protein 1 (HAP1) physically interacts with the huntingtin (HTT) protein. HAP1 functions in intracellular vesicle and	Expression levels of HAP1 are closely related to resistance against radiotherapy in glioblastoma and other cancer entities [67-71].	None	None

					organelle transport by linking HTT to motor proteins such as dynactin. In addition, HAP1 is important for autophagosomal degradation of proteins [65, 66].	HAP1 is also supposed to have functions in the pathogenicity of pancreatic cancer and breast cancer [66].		
TXNDC17	-	> 0.9	> 0.9	-	Thioredoxin Domain Containing protein 17 (TXNDC17) catalyzes the reduction of disulfides. TXNDC17 exhibits a peroxidase activity which is involved in the elimination of hydrogen peroxide from cells. TXNDC17 is also involved in Nuclear Factor κB (NF-κB) and Tumor Necrosis Factor alpha (TNFA) signaling [72].	Connections between TXNDC17 and glioblastoma or other cancer entities are not reported so far.<br><br>However, inhibition of protein disulfide isomerases in general is supposed to represent a promising approach for treatment of glioblastoma and other cancer entities [73].	None	None
NPEPPS	-	> 0.9	> 0.9	-	Puromycin-Sensitive Aminopeptidase M1 (NPEPPS) is a zinc metallopeptidase which hydrolyzes amino acids from the N-termini of substrate proteins such as enkephalins and Superoxide Dismutase 1 (SOD1) [74-76].	NPEPPS affects the migratory and the differentation behaviour of glioblastoma cells *in vitro* [77].	Puromycin (pan-inhibitor of M1 aminopeptidases)	None
KLHL11	-	> 0.9	> 0.9	-	Kelch-like famiy member protein 11 (KLHL11) is part of the cullin-RING-based BCR (BTB-CUL3-RBX1) ubiquitin ligase complex which ubiquitinates the NF-2-Related Factor 2 (NRF2), a core component of the oxidative stress response, upon oxidative stress [78, 79].	Connections between KLHL11 and glioblastoma or other cancer entities are not reported so far.<br><br>However, inhibition of NRF2/KEAP1 signaling per se represents a promising approach for cancer treatment [80-83].	No specific inhibitor of KLHL11 available so far.<br><br>Modulators (both, activators and inhibitors) of the NRF2/KEAP1 signaling hub are readily available and also trial-tested [84-86], e.g.<br><br>Resveratrol [87]<br>Curcumin [88]<br>Brusatol [89]<br>Oltipraz [90]<br>Omaveloxolone [91, 92]<br>ML385 [93]<br>ML334 [94]	None<br><br>Modulators of the NRF2/KEAP1 signaling hub are currently trial tested in patients with different malignancies [84-86], but so far not in glioblastoma patients.
PSMB3	-	> 0.9	> 0.9	-	The Proteasomal Subunit beta 3 (PSMB3) is a non-catalytic subunit of the 20S core proteasome. Thus, PSMB3 has a crucial function in the protein homeostasis of cells [95].	Connections between PSMB3 and glioblastoma or other cancer entities are not reported so far.<br><br>However, the proteasome as a target for cancer therapy is of great importance [96].	No specific inhibitor of PSMB3 available so far.<br><br>Inhibitors of the 20S/26S core/holo-proteasome are readily available and also trial-tested [96, 97], e.g.	None<br><br>Inhibitors of the 20S/26S core/holo-proteasome are currently trial tested in patients with different malignancies [96, 97], but so far not in glioblastoma patients.

							Bortezomib [98, 99]<br>Carfilzomib [100]<br>Celastrol [101, 102]<br>Delanzomib [103]<br>Ixazomib [104]<br>MG-132 [105]	Bortezomib is an FDA-approved drug for the treatment of multiple myeloma and of mantle cell lymphoma [106].
TGFBR2	< - 0.9	-	< - 0.9	-	Transforming Growth Factor (TGF) beta Receptor-2 (TGFBR2) is a subunit of the serine/threonine kinase associated receptor which, together with TGFBR1/ALK5, forms the TGF beta receptor (TGFBR) [107].	Expression of TGFBR2 correlates with expression levels of platelet derived growth factor receptor (PDFGR), a signature marker of the proneural molecular glioblastoma subtype, in glioblastoma cells [108]. In addition, TGFBR2 modulates TGF beta and Smad2/3-dependent signaling in glioblastoma cells [109].	No specific inhibitor of TGFBR2 available so far.<br><br>Inhibitors of TGFBR1 are readily available and also trial-tested [110], e.g.<br><br>Galunisertib [111]<br>Vactosertib [112, 113]<br>LY364947 [114]<br>LSKL [115]	None<br><br>Inhibitors of TGFBR1 are currently trial tested in patients with different malignancies including glioblastoma [110].<br><br>Galunisertib/LY2157299 was tested in combination with radiotherapy and TMZ in a phase 1B/2A trial on patients suffering from glioblastoma, but failed to improve clinical outcome (NCT01220271) [116].
MYNN	-	< - 0.9	< - 0.9	-	Myoneurin (MYNN) is a member of the protein family called BTB/POZ and zinc finger domain-containing proteins. This protein family is involved in the regulation of gene expression [117].	Connections between MYNN and glioblastoma or other cancer entities are not reported so far.	None	None
SYT14	-	< - 0.9	< - 0.9	-	Synaptotagmin-14 (SYT14) is a calcium-independent member of the synaptotagmin protein family which exhibits functions in synaptic transmission and exocytosis [118].	Connections between SYT14 and glioblastoma or other cancer entities are not reported so far.	CN110420328A (unpublished)	None
MED15	-	< - 0.9	< - 0.9	-	Mediator complex subunit 15 (MED15) is a central component of the mediator complex (MC) which acts as a co-activator in RNA polymerase II-dependent transcription [119].	Connections between MED15 and glioblastoma are not reported so far.<br><br>Alterations in expression of MED15 were detected in testis cancer, prostate cancer, and bladder cancer [120-123].	None	None
SYPL2	-	< - 0.9	< - 0.9	-	Synaptophysin-like 2 (SYPL2) mediates the communication between membranes of the T-tubular and the junctional sarcoplasmic reticulum (SR). As such, SYPL2 is involved in calcium homeostasis of the skeletal muscular system [124].	Connections between SYPL2 and glioblastoma or other cancer entities are not reported so far.<br><br>However, expression levels of Synaptophysin-like 1 (SYPL1) clearly correlate with clinical outcomes in cancer patients, e.g. in patients suffering from papillary thyroid cancer, and liver cancer [125, 126].	None	None
C1GALT1	-	< - 0.9	< - 0.9	-	Core 1 Glycoprotein-N-Acetyl-galactosamine-3-beta-Galactosyl-Transferase 1 (C1GALT1) catalyzes O-linked glycosylation of	Connections between C1GALT1 and glioblastoma are not reported so far.	None	None

					mucin-like proteins [127]. C1GALT1 has documented functions in angiogenesis, platelet production, and kidney development [128].	Yet, alterations in gene expression of C1GALT1 are associated with poor prognosis in head and neck cancer and gastric cancer [129, 130].		
POLR1F/ TWISTNB	-	< - 0.9	< - 0.9	-	RNA Polymerase 1 subunit F (POLR1F/TWISTNB) is part of the RNA polymerase 1 (POL1) complex which synthesizes ribosomal RNAs (rRNAs). POLR1F/TWISTNB regulates the 5'-3' RNA polymerase activity of the POL1 complex [131, 132].	Connections between POLR1F/TWISTNB and glioblastoma are not reported so far.<br><br>POLR1F/TWISTNB is overexpressed in lung cancer and testis cancer [133].	No specific inhibitor of POLR1F/TWISTNB available so far.<br><br>Inhibitors of POL1 transcription in general are readily available [134], e.g.<br><br>BMH-21 [135]<br>CX-3543 [136]<br>CX-5461 [137]	None
CFH	-	< - 0.9	< - 0.9	-	Complement Factor H (CFH) belongs to the Regulator of Complement Activation (RCA) cluster of genes [138]. The CFH glycoprotein is secreted into the blood where it regulates the activity of the complement system and thus, immune responses [139].	Overexpression of CFH in glioblastoma cells as the result of overexpression of non-metabolic indoleamine 2,3-dioxygenase 1 (IDO1) suppresses anti-tumor immune responses thereby impairing survival in syngeneic mouse models of glioblastoma [140]. Furthermore, CFH promotes the progression of glioblastoma cells by affecting AKT1 and miR-149 [141].<br><br>Finally, CFH expression is accelerated in ovarian cancer and lung cancer [142, 143].	None	None

Supplementary Table 5: CGC genes with overlapping positive (R ≥ 0.7) correlation with inherent therapy resistance in human glioblastoma cells, for which drugs are readily available

Name	SSIR	FIR	COMBI	TMZ	General Information	Connection to Glioblastoma	Inhibitors	Clinical Trials
AR	≥ 0.7	≥ 0.7	≥ 0.7	-	Androgen Receptor (AR) is a nuclear receptor activated by androgenic hormones like testosterone and dihydrotestosterone. AR acts as an activator of transcription regulating the expression of androgen-responsive genes like KLK2 and KLK3 [144]. As such, AR exhibits functions in the development and the maintenance of the reproductive system as well as in the cardiovascular, the musculoskeletal, and the haematopoietic system [145, 146].	Overexpression of AR has been associated with reduced survival in glioblastoma patients [147-152]. Activation of AR signaling in glioblastoma cells accelerates their proliferation, clonogenic ability, migratory behaviour, invasiveness, and therapy resistance (both to radiotherapy and TMZ) [151-158], and these effects can be reversed by AR-targeting approaches [152, 156-158].	AR inhibitors are readily available and also trial-tested [146, 159-161], e.g.<br><br>Apalutamide/ARN-509 [162]<br>Bicalutamide/ICI-176334 [163, 164]<br>Darolutamide/OMD-201 [165]<br>Enzalutamide/MVD3100 [166]<br>Flutamide/SCH-13521 [167]<br>Nilutamide/RU23908 [168, 169]<br>Cyproterone acetate [170]<br>ALZ003 [152]	AR inhibitors are currently trial-tested in patients with different malignancies focussing on prostate cancer [146, 159-161]. No trials in glioblastoma patients so far.<br><br>Several AR inhibitors such as Apalutamide and Darolutamide are FDA-approved for treatment of prostate cancer [171].
STAT5b	≥ 0.7	≥ 0.7	≥ 0.7	-	Signal Transducer and Activator of Transcription 5b (STAT5b) induces the transcription of target genes such as FOXP3 and IL2RA [172] upon its activation by the Janus Kinase (JAK) in response to extracellular signals (e.g. IL2, IL4, CSF1) [173].	STAT5b has been associated with poor prognosis in glioblastoma patients [174]. Pro-malignant signaling mediated by the Epidermal Growth Factor Receptor variant III (EGFR vIII)/STAT5 axis was shown to contribute significantly to survival and migration of glioblastoma cells [175, 176], and inhibition of STAT5 suppressed the proliferation, invasion, and stemness of glioblastoma cells *in vitro* and *in vivo* [177, 178].	No specific inhibitor of STAT5b available so far.<br><br>However, pan-STAT5 inhibitors are readily available and also trial-tested [179], e.g.<br><br>STAT5-IN-1 [180] (pan-STAT5 inhibitor)<br>IQDMA [181] (pan-STAT5 inhibitor)<br>Pimozide [182] (pan-STAT5 inhibitor)<br>AC-4-130 [183] (pan-STAT5 inhibitor)<br>IST5-002 [184] (pan-STAT5 inhibitor)<br>BD750 [185] (pan-STAT5 inhibitor, co-inhibits JAK3)	Pan-STAT5 inhibitors are currently trial tested in patients with different malignancies focussing on the haematopoetic system [179]. No trials in glioblastoma patients so far.

MAP2K4	≥ 0.7	≥ 0.7	≥ 0.7	-	Dual specificity Mitogen-Activated Kinase Kinase 4 (MAP2K4/MEK4/ MKK4) is a part of the MAPK (RAF-MEK-ERK) and Stress-Activated Protein (SAP) Kinase/c-Jun N-terminal Kinase (JNK) signaling pathway network [27].	MAP2K4 exhibits tumor suppressing functions by decelerating tumor progression and metastasis formation [28-33]. MAP2K4 is frequently mutated in cancer, and mutations of MAP2K4 exhibit proto-oncogenic potentials [34-40]. Mutations of MAP2K4 often conincide with resistances to MAPK-targeting therapies [41].<br><br>MAP2K4 contributes to glioblastoma progression by promoting the self-renewal of GSCs [42]. This effect can be reverted by Genistein [42]. Genistein synergizes with radiotherapy and TMZ *in vitro* [44, 45]. Expression of miR-744, a MAP2K4-targeting miRNA, interferes with migration and invasiveness of glioblastoma cells *in vitro* [46].	MEK4 inhibitor-1 [47]<br>MEK4 inhibitor-2 [47]<br>3-Arylimidazoles [48]<br>BSJ-04-122 [49]<br>(co-inhibits MEK7)<br>HRX-0215 (unpublished)<br><br>Genistein/NPI031L [43] (protein tyrosine kinase (PTK) inhibitor)	Genistein was tested in multiple clinical trials in patients with different diseases including different malignancies, but not in glioblastoma patients so far [50].<br><br>Currently, Genistein is tested in combination with systemic chemotherapy in a randomized trial on pediatric patients either suffering from lymphomas or from solid tumors including pediatric gliomas (NCT02624388).

Supplementary Table 6: Intersect leading edge genes for which drugs are readily available

**Leading edge genes at the SSIR and FIR intersection of the reactive oxygen species (ROS) pathway gene set for which inhibitors are readily available**				
**Name**	**General Information**	**Connections to Glioblastoma**	**Inhibitors**	**Clinical Trials**
G6PD	Glucose-6-phosphate-1-dehydrogenase (G6PD) is an enzyme of the pentose phosphate pathway [186] whose main function is the production of NADPH, a key electron donor in defense against oxidizing agents, and reductive biosynthetic reactions [60, 187, 188].	G6PD is essential for protection from oxidative stress in GSCs [189]. Overexpression of G6PD correlates with reduced survival, and poor prognosis in glioblastoma patients, and with increased proliferation and therapy resistance in glioblastoma cell lines [190-192].	6-Aminonicotinamide [193]<br>RRx-001 [194-196]<br>(epigenetically active [197])<br>Polydatin [198]<br>G6PDi-1 [199]<br>Wedelolactone [200]	RRx-001 is currently tested in a phase I trial in combination with fractionated radiotherapy (30x 2 Gy) and TMZ in patients suffering from primary glioblastoma or anaplastic glioma (NCT02871843).<br>.
GCLC	Glutamate-Cysteine Ligase catalytic subunit (GCLC) catalyzes the ligation of L-cysteine with L-glutamate, a rate-limiting step in the synthesis of gluthathione (GSH) [201, 202]. Thus, GCLC is essential for protection from oxidative stress and for intracellular redox homeostasis [202].	Expression of GCLC in glioblastoma cells is regulated by CD147 [203, 204], and isocitrate dehydrogenase 1 (IDH1) [205, 206].	L-Buthionine-Sulfoximine (L-BSO) [207]<br>2-Deoxy-d-ribose [208]<br>$NaAsO_2$ [209]	L-BSO was tested in a phase I trial on pediatric neuroblastoma patients (NCT00005835) [210, 211]. No trial data for glioblastoma so far.
TXNRD1	Thioredoxin Reductase 1 (TXNRD1) is a selenocysteine-containing flavoenzyme of the pyridine nucleotide-disulfide oxidoreductase family. TXNRD1 is an integral component of the thioredoxin reductive system (TRX) which eliminates reactive oxygen species (ROS) thereby ensuring redox homoeostasis in cells [212, 213].	Deregulations in expression of thioredoxin reductases (TXNRs) including TXNRD1 are associated with malignancy, progression, and angiogenesis in glioblastoma [214-217]. Interference with TXNRD1/TXN function sensitizes glioblastoma cells to chemotherapy and to radiotherapy [218-222].	Auranofin [223-226]<br>(pan-TXNR inhibitor)<br>Curcumin [227]<br>Diffractaic acid [228]<br>Ethaselene [229]<br>Laromustine [230]<br>(pan-TXNR inhibitor)<br>Myricetin [231]<br>Piperlongumine [232]<br>Santamarine [233]<br>TRi-1/HUN20688 [234]<br>TrxR1-5 [218]<br>TrxR1-6 [218]<br>IP-Se-06 [235]	Ethaselene is tested in a phase Ic trial (NCT02166242) in non-small cell lung cancer patients. No trial data on glioblastoma so far.
TXN	Thioredoxin (TXN) acts as a homodimer and is active in S-nitrosylation of cysteines, an integral step in response to intracellular nitric oxides [212, 213].	Deregulation of TXN expression is reported for many cancers including glioblastoma [215, 236, 237]. Interference with TXN function sensitizes glioblastoma cells to radiotherapy [238].	Arsenic trioxide [239]<br>Diallyl trisulfite [238]<br>Pleurotin [240]<br>PX-12/DB05448 [240]<br>Diallyl trisulfite [238]	PX-12 was tested in several trials on different malignancies [241-243], but not on glioblastoma so far.
NDUFB4	NADH:Ubiquinone oxidoreductase subunit B4 (NDUFB4) is a non-catalytic subunit of the NADH:Ubiquinone oxidoreductase enzyme complex (complex I) of the mitochondrial electron transport chain [244].	NDUFB4, alongside with other genes of the ATP generating system, is upregulated in different glioblastoma subtypes [245]. Deregulations of NDUFB4 are a hallmark of many treatment-resistant cancers [246-248].	No specific inhibitor of NDUFB4 available so far.<br><br>Inhibitors of the oxidative phosphorylation (OXPHOS)-related respiratory chain complex I are readily available and also trial-tested, e.g.<br><br>Metformin [249-252]<br>Intervenolin [253, 254]<br>Gboxin [255]<br>Olaparib [256]<br>EVT-701 [257]	OXPHOS inhibitors, e.g. ME-143, ME-344, IACS-010759, and BAY-87-2243 are trial tested in different types of malignancy [258, 260, 265], but so far not in glioblastoma.

			ME-143 [258, 259]<br>ME-344 [259, 260]<br>IACS-010759 [261]<br>BAY-87-2243 [262, 263]<br><br>Multiple natural products, e.g. rotenoids and piericidins, as well as synthetic compounds, e.g. insecticides [264].	
STK25	Serine/Threonine kinase 25 (STK25) is a member of the germinal centre kinase III (GCK III) subfamily belonging to the sterile 20 kinase superfamily. STK25 is involved in serine/threonine liver kinase B1 (LKB1) signaling, regulating neuronal polarization and morphology of the Golgi apparatus. STK25 is translocated from the Golgi apparatus to nuclei in response to anoxia, and also plays a role in the regulation of cell death [266, 267].	Connections between STK25 and glioblastoma are not reported so far.<br><br>STK25 exhibits tumor-suppressive functions as it directly activates the tumor-suppressing Hippo signaling pathway [268]. STK25 also inhibits glycolysis [269]. However, tumor-promoting functions of STK25 are reported as well, particularly in liver cancer [270, 271].	None	None
GSR	Gluththione Disulfide Reductase (GSR) is a homodimeric flavoprotein and member of the class-I pyridine nucleotide-disulfide oxidoreductase family. GSR is a core enzyme of the antioxidant defense, as it reduces oxidized glutathione disulfide (GSSG) to its sulfhydryl form GSH [201, 202].	GSR mediates drug resistance in glioblastoma cells via its function to regulate redox homeostasis [272]. GSR is deregulated in several cancer entities including lung cancer and liver cancer [273-275].	GSH [276]<br>2-AAPA [277]<br>LCS3 [278]<br>(co-inhibits TXNRD1)	None
ABCC1	ATP Binding Cassette subfamily C member 1 (ABCC1) is a member of the superfamily of ATP-Binding Cassette (ABC) transporters. ABCC1 is involved in drug resistance, functioning as a multispecific organic anion transporter with a substrate range encompassing oxidized GSH, cysteinyl leukotrienes, activated aflatoxins, glucuronides, and sulfate conjugates of steroid hormones [279, 280].	ABCC1 alongside with other ABC transporters is overexpressed in many cancer entities including glioblastoma [281, 282]. ABCC1 contributes to therapy resistance by eliminating therapeutic agents from cancer cells. In glioblastoma, several miRNAs that target ABCC1 have been identified, and their expression levels determine the degree of therapy resistance in these tumors [283-287].	MK-571 [288]<br>Reversan [289]<br>Thienopyrimidines [290]	None
**Leading edge genes at the SSIR and TMZ intersection of the mammalian target of rapamycin complex 1 (mTORC1) pathway gene set for which inhibitors are readily available**				
**Name**	**General Information**	**Connections to Glioblastoma**	**Inhibitors**	**Clinical Trials**
PSMG1	Proteasome assembly chaperone 1 (PSMG1) dimerizes with PSMG2 to form a chaperone complex with molecular adaptor activity that is crucial for the assembly of the 20S core proteasome [291].	PSMG1 as well as other proteasomal components are frequently deregulated in cancers [292-295], including glioblastoma [296].	No specific inhibitor of PSMG1 available so far.<br><br>Inhibitors of the 20S/26S core/holo-proteasome are readily available and trial-tested [96, 97], e.g.<br><br>Bortezomib [98, 99]<br>Carfilzomib [100]<br>Celastrol [101, 102]<br>Delanzomib [103]<br>Ixazomib [104]<br>MG-132 [105]	None<br><br>Inhibitors of the 20S/26S core/holo-proteasome are currently trial tested on different malignancies [96, 97], but not on glioblastoma.<br><br>Bortezomib/Velcade is an FDA-approved drug for the treatment of multiple myeloma and mantle cell lymphoma [106].

PSMA4	Proteasome 20S subunit alpha 4 (PSMA4) constitutes a core subunit of the 20S core proteasome, thus playing an important role in protein homeostasis [297].	Connections between PSMA4 and glioblastoma are not reported so far.<br><br>However, polymorphisms of PSMA4 have been associated with increased susceptibility to lung cancer [298].	No specific inhibitor of PSMG1 available so far.<br><br>Inhibitors of the 20S/26S core/holo-proteasome are readily available and trial-tested [96, 97], e.g.<br><br>Bortezomib [98, 99]<br>Carfilzomib [100]<br>Celastrol [101, 102]<br>Delanzomib [103]<br>Ixazomib [104]<br>MG-132 [105]	None<br><br>Inhibitors of the 20S/26S core/holo-proteasome are currently trial tested on different malignancies [96, 97], but not on glioblastoma.<br><br>Bortezomib/Velcade is an FDA-approved drug for the treatment of multiple myeloma and mantle cell lymphoma [106].
HPRT1	Hypoxanthine phosphoribosyltransferase 1 (HPRT1) is an enzyme that catalyzes the conversions of hypoxanthine to inosine monophosphate and of guanine to guanosine monophosphate. Thus, HPRT1 plays a crucial role in purine salvage pathway-dependent synthesis of purine nucleotides [299, 300].	Connections between HPRT1 and glioblastoma are not reported so far.<br><br>HPRT1 is upregulated in various cancer entities, resulting in worsened prognoses and increased levels of therapy resistance [300-303].	None	None
RRM2	Ribonucleotide Reductase regulatory subunit M2 (RRM2) is one of two subunits that form ribonucleotide reductase. RRM2 catalyzes the conversion of ribonucleotides into desoxyribonucleotides [304].	RRM2 is overexpressed in glioblastoma [305], and its overexpression correlates with reduced patient survival and increased resistance to therapy [305-308]. Similar data are reported for other cancer entities [309-315]	Hydroxyurea<br>COH29 [316]<br>Osalmid [317]<br>Pectolinarigenin [307]<br>4-Hydroxysalicylanilide [318]<br>(for review see [312])	4-Hydroxysalicylanilide was tested in a phase I trial on patients with multiple myeloma (NCT03670173) [318]. No trial data on glioblastoma so far.
SLC7A11	Solute Carrier family 7 member 11 (SLC7A11) is part of a heteromeric anionic amino acid transporter with specificity for cysteine and glutamate. In this system called Xc(-), the anionic form of cysteine is imported into cells in exchange for glutamate [319-321].	SLC7A11 is frequently overexpressed in glioblastoma [322], and its overexpression correlates with reduced survival and poor prognosis [308, 322]. Mechanistically, overexpression of SLC7A11 increases the stem cell-like properties of glioblastoma cells [323]. Similar findings were published for other cancer entities [319, 324].	No specific inhibitor of SLC7A11 available so far.<br><br>Inhibitors of the Xc(-) system are readily available [96, 97], e.g.<br><br>Erastin [325]<br>Sulfasalazine [326]<br>Imidazole ketone erastin [327]<br>HG106 [328]	None
SLC1A5/ASCT2	Solute Carrier family 1 member 5 (SLC1A5/ASCT2) is a sodium-dependent neutral amino acid transporter with high specifity for glutamine. As such, SLC1A5 plays an important role in the redox homeostasis in cells [329].	SLC1A5/ASCT2 contributes to tumorigenesis and tumor progression in several cancer entities including head-and-neck cancer, stomach cancer and liver cancer [330-335]. Variants of SLC1A5 were shown to reprogram cancer cells thereby facilitating therapy resistances [336]. SLC1A5/ASCT2 is also overexpressed in astrocytomas and glioblastomas [337, 338].	L-γ-Glutamyl-p-nitroanilide (GPNA) [339]<br>V9302 [340]<br>Sulfonamide/sulfonic acid ester scaffolds [341]<br>Lc-proline biphenyl esters [342]	None

References

1. Szymczak-Kulus, K., et al., *Human Gb3/CD77 synthase produces P1 glycotope-capped N-glycans, which mediate Shiga toxin 1 but not Shiga toxin 2 cell entry.* J Biol Chem, 2021. **296**: p. 100299.

2. Piazzesi, A., S.Y. Afsar, and G. van Echten-Deckert, *Sphingolipid metabolism in the development and progression of cancer: one cancer's help is another's hindrance.* Mol Oncol, 2021. **15**(12): p. 3256-3279.

3. Jacob, F., et al., *Transition of Mesenchymal and Epithelial Cancer Cells Depends on alpha1-4 Galactosyltransferase-Mediated Glycosphingolipids.* Cancer Res, 2018. **78**(11): p. 2952-2965.

4. Pellegrini, L., *The Pol alpha-primase complex.* Subcell Biochem, 2012. **62**: p. 157-69.

5. Pollok, S., et al., *Regulation of eukaryotic DNA replication at the initiation step.* Biochem Soc Trans, 2003. **31**(Pt 1): p. 266-9.

6. Ercilla, A., et al., *Physiological Tolerance to ssDNA Enables Strand Uncoupling during DNA Replication.* Cell Rep, 2020. **30**(7): p. 2416-2429 e7.

7. O'Donnell, M., L. Langston, and B. Stillman, *Principles and concepts of DNA replication in bacteria, archaea, and eukarya.* Cold Spring Harb Perspect Biol, 2013. **5**(7).

8. Starokadomskyy, P., et al., *DNA polymerase-alpha regulates the activation of type I interferons through cytosolic RNA:DNA synthesis.* Nat Immunol, 2016. **17**(5): p. 495-504.

9. Yamaguchi, A., et al., *DNA polymerase alpha positive-cell rate in colorectal cancer and its relationship to prognosis.* Br J Cancer, 1992. **65**(3): p. 421-4.

10. Tateishi, M., et al., *DNA polymerase alpha as an independent prognostic parameter in non-small cell lung cancer--an immunohistochemical study.* Eur J Surg Oncol, 1994. **20**(4): p. 461-6.

11. Heitzer, E. and I. Tomlinson, *Replicative DNA polymerase mutations in cancer.* Curr Opin Genet Dev, 2014. **24**: p. 107-13.

12. Berdis, A.J., *Inhibiting DNA Polymerases as a Therapeutic Intervention against Cancer.* Front Mol Biosci, 2017. **4**: p. 78.

13. Han, T., et al., *The antitumor toxin CD437 is a direct inhibitor of DNA polymerase alpha.* Nat Chem Biol, 2016. **12**(7): p. 511-5.

14. Abdel-Samad, R., et al., *Mechanism of action of the atypical retinoid ST1926 in colorectal cancer: DNA damage and DNA polymerase alpha.* Am J Cancer Res, 2018. **8**(1): p. 39-55.

15. El Hajj, H., et al., *Preclinical efficacy of the synthetic retinoid ST1926 for treating adult T-cell leukemia/lymphoma.* Blood, 2014. **124**(13): p. 2072-80.

16. Dallavalle, S., et al., *Antitumor activity of novel POLA1-HDAC11 dual inhibitors.* Eur J Med Chem, 2022. **228**: p. 113971.

17. Park, S.Y. and X. Guo, *Adaptor protein complexes and intracellular transport.* Biosci Rep, 2014. **34**(4).

18. Freire-Beneitez, V., et al., *Elucidation of the BMI1 interactome identifies novel regulatory roles in glioblastoma.* NAR Cancer, 2021. **3**(1): p. zcab009.

19. Danan-Gotthold, M., et al., *Identification of recurrent regulated alternative splicing events across human solid tumors.* Nucleic Acids Res, 2015. **43**(10): p. 5130-44.

20. Rangel, R., et al., *Identification of New Tumor Suppressor Genes in Triple-Negative Breast Cancer.* Cancer Res, 2017. **77**(15): p. 4089-4101.

21. Beautrait, A., et al., *A new inhibitor of the beta-arrestin/AP2 endocytic complex reveals interplay between GPCR internalization and signalling.* Nat Commun, 2017. **8**: p. 15054.

22. Yang, S. and N.B. Hecht, *Translin associated protein X is essential for cellular proliferation.* FEBS Lett, 2004. **576**(1-2): p. 221-5.

23. Wang, Y., et al., *Development of a nomogram for prognostic prediction of lower-grade glioma based on alternative splicing signatures.* Cancer Med, 2020. **9**(24): p. 9266-9281.

24. Yu, Y., et al., *ZZEF1 is a Histone Reader and Transcriptional Coregulator of Kruppel-Like Factors.* J Mol Biol, 2021. **433**(2): p. 166722.

25. Giunti, L., et al., *Genome-wide copy number analysis in pediatric glioblastoma multiforme.* Am J Cancer Res, 2014. **4**(3): p. 293-303.

26. Joo, J.E., et al., *Heritable DNA methylation marks associated with susceptibility to breast cancer.* Nat Commun, 2018. **9**(1): p. 867.

27. Cuenda, A., *Mitogen-activated protein kinase kinase 4 (MKK4).* Int J Biochem Cell Biol, 2000. **32**(6): p. 581-7.

28. Davis, S.J., et al., *Analysis of the mitogen-activated protein kinase kinase 4 (MAP2K4) tumor suppressor gene in ovarian cancer.* BMC Cancer, 2011. **11**: p. 173.

29. Ahn, Y.H., et al., *Map2k4 functions as a tumor suppressor in lung adenocarcinoma and inhibits tumor cell invasion by decreasing peroxisome proliferator-activated receptor gamma2 expression.* Mol Cell Biol, 2011. **31**(21): p. 4270-85.

30. Spillman, M.A., et al., *Regulation of the metastasis suppressor gene MKK4 in ovarian cancer.* Gynecol Oncol, 2007. **105**(2): p. 312-20.

31. Robinson, V.L., et al., *Mitogen-activated protein kinase kinase 4/c-Jun NH2-terminal kinase kinase 1 protein expression is subject to translational regulation in prostate cancer cell lines.* Mol Cancer Res, 2008. **6**(3): p. 501-8.

32. Yamada, S.D., et al., *Mitogen-activated protein kinase kinase 4 (MKK4) acts as a metastasis suppressor gene in human ovarian carcinoma.* Cancer Res, 2002. **62**(22): p. 6717-23.

33. Kim, H.L., et al., *Mitogen-activated protein kinase kinase 4 metastasis suppressor gene expression is inversely related to histological pattern in advancing human prostatic cancers.* Cancer Res, 2001. **61**(7): p. 2833-7.

34. Chae, K.S., et al., *Expression and mutation analyses of MKK4, a candidate tumour suppressor gene encoded by chromosome 17p, in human gastric adenocarcinoma.* Eur J Cancer, 2002. **38**(15): p. 2048-57.

35. Su, G.H., et al., *Mutation rate of MAP2K4/MKK4 in breast carcinoma.* Hum Mutat, 2002. **19**(1): p. 81.

36. Xin, W., et al., *MAP2K4/MKK4 expression in pancreatic cancer: genetic validation of immunohistochemistry and relationship to disease course.* Clin Cancer Res, 2004. **10**(24): p. 8516-20.

37. Huang, C., et al., *Overexpression of mitogen-activated protein kinase kinase 4 and nuclear factor-kappaB in laryngeal squamous cell carcinoma: a potential indicator for poor prognosis.* Oncol Rep, 2009. **22**(1): p. 89-95.

38. Finegan, K.G. and C. Tournier, *The mitogen-activated protein kinase kinase 4 has a pro-oncogenic role in skin cancer.* Cancer Res, 2010. **70**(14): p. 5797-806.

39. Pavese, J.M., et al., *Mitogen-activated protein kinase kinase 4 (MAP2K4) promotes human prostate cancer metastasis.* PLoS One, 2014. **9**(7): p. e102289.

40. Whitmarsh, A.J. and R.J. Davis, *Role of mitogen-activated protein kinase kinase 4 in cancer.* Oncogene, 2007. **26**(22): p. 3172-84.

41. Xue, Z., et al., *MAP3K1 and MAP2K4 mutations are associated with sensitivity to MEK inhibitors in multiple cancer models.* Cell Res, 2018. **28**(7): p. 719-729.

42. Chen, X., et al., *Activation of JNK and p38 MAPK Mediated by ZDHHC17 Drives Glioblastoma Multiforme Development and Malignant Progression.* Theranostics, 2020. **10**(3): p. 998-1015.

43. Akiyama, T., et al., *Genistein, a specific inhibitor of tyrosine-specific protein kinases.* J Biol Chem, 1987. **262**(12): p. 5592-5.

44. Liu, X., et al., *Genistein inhibits radiation-induced invasion and migration of glioblastoma cells by blocking the DNA-PKcs/Akt2/Rac1 signaling pathway.* Radiother Oncol, 2021. **155**: p. 93-104.

45. Atefeh, Z., et al., *Combination Treatment of Glioblastoma by Low-Dose Radiation and Genistein.* Curr Radiopharm, 2016. **9**(3): p. 258-263.

46. Hubner, M., et al., *Intronic miR-744 Inhibits Glioblastoma Migration by Functionally Antagonizing Its Host Gene MAP2K4.* Cancers (Basel), 2018. **10**(11).

47. Kwong, A.J., et al., *Rational Design, Optimization, and Biological Evaluation of Novel MEK4 Inhibitors against Pancreatic Adenocarcinoma.* ACS Med Chem Lett, 2021. **12**(10): p. 1559-1567.

48. Deibler, K.K., et al., *Synthesis and Biological Evaluation of 3-Arylindazoles as Selective MEK4 Inhibitors.* ChemMedChem, 2019. **14**(6): p. 615-620.

49. Jiang, J., et al., *Discovery of Covalent MKK4/7 Dual Inhibitor.* Cell Chem Biol, 2020. **27**(12): p. 1553-1560 e8.

50. Mathew, S. and C.G. Vazhappilly, *Recent pharmacological advances on genistein in clinical trials.* EXCLI J, 2020. **19**: p. 1120-1123.

51. Mardian, E.B., R.M. Bradley, and R.E. Duncan, *The HRASLS (PLA/AT) subfamily of enzymes.* J Biomed Sci, 2015. **22**: p. 99.

52. Uyama, T., et al., *Characterization of the human tumor suppressors TIG3 and HRASLS2 as phospholipid-metabolizing enzymes.* Biochim Biophys Acta, 2009. **1791**(12): p. 1114-24.

53. Chen, C., et al., *Identification of key genes in glioblastoma-associated stromal cells using bioinformatics analysis.* Oncol Lett, 2016. **11**(6): p. 3999-4007.

54. Zhou, J., et al., *Activity-Based Protein Profiling Identifies alpha-Ketoamides as Inhibitors for Phospholipase A2 Group XVI.* ACS Chem Biol, 2019. **14**(2): p. 164-169.

55. Zhou, J., et al., *Structure-Activity Relationship Studies of alpha-Ketoamides as Inhibitors of the Phospholipase A and Acyltransferase Enzyme Family.* J Med Chem, 2020. **63**(17): p. 9340-9359.

56. Wu, L., et al., *Regulation of PLC-mediated signalling in vivo by CDP-diacylglycerol synthase.* Nature, 1995. **373**(6511): p. 216-22.

57. Shen, H. and W. Dowhan, *Regulation of phospholipid biosynthetic enzymes by the level of CDP-diacylglycerol synthase activity.* J Biol Chem, 1997. **272**(17): p. 11215-20.

58. Yeh, K.T., et al., *Methylation Inactivates Expression of CDP-diacylglycerol Synthase 1 (CDS1) in Hepatocellular Carcinoma.* Cancer Genomics Proteomics, 2006. **3**(3-4): p. 231-238.

59. Wang, H., et al., *Integrative analysis of somatic mutations and differential expression profiles in glioblastoma based on aging acceleration.* Int J Clin Exp Pathol, 2021. **14**(5): p. 582-595.

60. Kucharzewska, P., H.C. Christianson, and M. Belting, *Global profiling of metabolic adaptation to hypoxic stress in human glioblastoma cells.* PLoS One, 2015. **10**(1): p. e0116740.

61. Monne, M., et al., *The mitochondrial oxoglutarate carrier: from identification to mechanism.* J Bioenerg Biomembr, 2013. **45**(1-2): p. 1-13.

62. Lee, J.S., et al., *Loss of SLC25A11 causes suppression of NSCLC and melanoma tumor formation.* EBioMedicine, 2019. **40**: p. 184-197.

63. Pan, G., et al., *SLC25A11 serves as a novel prognostic biomarker in liver cancer.* Sci Rep, 2020. **10**(1): p. 9871.

64. Buffet, A., et al., *Germline Mutations in the Mitochondrial 2-Oxoglutarate/Malate Carrier SLC25A11 Gene Confer a Predisposition to Metastatic Paragangliomas.* Cancer Res, 2018. **78**(8): p. 1914-1922.

65. Wu, L.L. and X.F. Zhou, *Huntingtin associated protein 1 and its functions.* Cell Adh Migr, 2009. **3**(1): p. 71-6.

66. Zhao, X., et al., *Biological functions and potential therapeutic applications of huntingtin-associated protein 1: progress and prospects.* Clin Transl Oncol, 2022. **24**(2): p. 203-214.

67. Wu, J., et al., *HAP1 gene expression is associated with radiosensitivity in breast cancer cells.* Biochem Biophys Res Commun, 2015. **456**(1): p. 162-6.

68. Prieto-Alamo, M.J. and F. Laval, *Overexpression of the human HAP1 protein sensitizes cells to the lethal effect of bioreductive drugs.* Carcinogenesis, 1999. **20**(3): p. 415-9.

69. Herring, C.J., et al., *Expression levels of the DNA repair enzyme HAP1 do not correlate with the radiosensitivities of human or HAP1-transfected rat cell lines.* Br J Cancer, 1999. **80**(7): p. 940-5.

70. Amen, A.M., et al., *Cancer-specific loss of TERT activation sensitizes glioblastoma to DNA damage.* Proc Natl Acad Sci U S A, 2021. **118**(13).

71. Walker, L.J., et al., *A role for the human DNA repair enzyme HAP1 in cellular protection against DNA damaging agents and hypoxic stress.* Nucleic Acids Res, 1994. **22**(23): p. 4884-9.

72. Chatterji, A. and R. Sengupta, *Cellular S-denitrosylases: Potential role and interplay of Thioredoxin, TRP14, and Glutaredoxin systems in thiol-dependent protein denitrosylation.* Int J Biochem Cell Biol, 2021. **131**: p. 105904.

73. Kyani, A., et al., *Discovery and Mechanistic Elucidation of a Class of Protein Disulfide Isomerase Inhibitors for the Treatment of Glioblastoma.* ChemMedChem, 2018. **13**(2): p. 164-177.

74. Kudo, L.C., et al., *Puromycin-sensitive aminopeptidase (PSA/NPEPPS) impedes development of neuropathology in hPSA/TAU(P301L) double-transgenic mice.* Hum Mol Genet, 2011. **20**(9): p. 1820-33.

75. Ren, G., et al., *Cu, Zn-superoxide dismutase 1 (SOD1) is a novel target of Puromycin-sensitive aminopeptidase (PSA/NPEPPS): PSA/NPEPPS is a possible modifier of amyotrophic lateral sclerosis.* Mol Neurodegener, 2011. **6**: p. 29.

76. Agrawal, N. and M.A. Brown, *Genetic associations and functional characterization of M1 aminopeptidases and immune-mediated diseases.* Genes Immun, 2014. **15**(8): p. 521-7.

77. Verano-Braga, T., et al., *SuperQuant-assisted comparative proteome analysis of glioblastoma subpopulations allows for identification of potential novel therapeutic targets and cell markers.* Oncotarget, 2018. **9**(10): p. 9400-9414.

78. Dhanoa, B.S., et al., *Update on the Kelch-like (KLHL) gene family.* Hum Genomics, 2013. **7**: p. 13.

79. Panieri, E., et al., *NRF2 and Mitochondrial Function in Cancer and Cancer Stem Cells.* Cells, 2022. **11**(15).

80. Shahcheraghi, S.H., et al., *The Role of NRF2/KEAP1 Pathway in Glioblastoma: Pharmacological Implications.* Med Oncol, 2022. **39**(5): p. 91.

81. Kanamori, M., et al., *Activation of the NRF2 pathway and its impact on the prognosis of anaplastic glioma patients.* Neuro Oncol, 2015. **17**(4): p. 555-65.

82. Olivier, C., et al., *Drug Resistance in Glioblastoma: The Two Faces of Oxidative Stress.* Front Mol Biosci, 2020. **7**: p. 620677.

83. Godoy, P., et al., *Targeting NRF2, Regulator of Antioxidant System, to Sensitize Glioblastoma Neurosphere Cells to Radiation-Induced Oxidative Stress.* Oxid Med Cell Longev, 2020. **2020**: p. 2534643.

84. Robledinos-Anton, N., et al., *Activators and Inhibitors of NRF2: A Review of Their Potential for Clinical Development.* Oxid Med Cell Longev, 2019. **2019**: p. 9372182.

85. Yagishita, Y., et al., *Current Landscape of NRF2 Biomarkers in Clinical Trials.* Antioxidants (Basel), 2020. **9**(8).

86. Pouremamali, F., et al., *An update of Nrf2 activators and inhibitors in cancer prevention/promotion.* Cell Commun Signal, 2022. **20**(1): p. 100.

87. Alavi, M., et al., *Resveratrol mediates its anti-cancer effects by Nrf2 signaling pathway activation.* Cancer Cell Int, 2021. **21**(1): p. 579.

88. Ashrafizadeh, M., et al., *Curcumin Activates the Nrf2 Pathway and Induces Cellular Protection Against Oxidative Injury.* Curr Mol Med, 2020. **20**(2): p. 116-133.

89. Olayanju, A., et al., *Brusatol provokes a rapid and transient inhibition of Nrf2 signaling and sensitizes mammalian cells to chemical toxicity-implications for therapeutic targeting of Nrf2.* Free Radic Biol Med, 2015. **78**: p. 202-12.

90. Ramos-Gomez, M., et al., *Sensitivity to carcinogenesis is increased and chemoprotective efficacy of enzyme inducers is lost in nrf2 transcription factor-deficient mice.* Proc Natl Acad Sci U S A, 2001. **98**(6): p. 3410-5.

91. Reisman, S.A., et al., *Topical application of the synthetic triterpenoid RTA 408 activates Nrf2 and induces cytoprotective genes in rat skin.* Arch Dermatol Res, 2014. **306**(5): p. 447-54.

92. Probst, B.L., et al., *RTA 408, A Novel Synthetic Triterpenoid with Broad Anticancer and Anti-Inflammatory Activity.* PLoS One, 2015. **10**(4): p. e0122942.

93. Singh, A., et al., *Small Molecule Inhibitor of NRF2 Selectively Intervenes Therapeutic Resistance in KEAP1-Deficient NSCLC Tumors.* ACS Chem Biol, 2016. **11**(11): p. 3214-3225.

94. Hu, L., et al., *Discovery of a small-molecule inhibitor and cellular probe of Keap1-Nrf2 protein-protein interaction.* Bioorg Med Chem Lett, 2013. **23**(10): p. 3039-43.

95. Bard, J.A.M., et al., *Structure and Function of the 26S Proteasome.* Annu Rev Biochem, 2018. **87**: p. 697-724.

96. Manasanch, E.E. and R.Z. Orlowski, *Proteasome inhibitors in cancer therapy.* Nat Rev Clin Oncol, 2017. **14**(7): p. 417-433.

97. Adams, J., *Proteasome inhibition: a novel approach to cancer therapy.* Trends Mol Med, 2002. **8**(4 Suppl): p. S49-54.

98. Hideshima, T., et al., *The proteasome inhibitor PS-341 inhibits growth, induces apoptosis, and overcomes drug resistance in human multiple myeloma cells.* Cancer Res, 2001. **61**(7): p. 3071-6.

99. LeBlanc, R., et al., *Proteasome inhibitor PS-341 inhibits human myeloma cell growth in vivo and prolongs survival in a murine model.* Cancer Res, 2002. **62**(17): p. 4996-5000.

100. Kuhn, D.J., et al., *Potent activity of carfilzomib, a novel, irreversible inhibitor of the ubiquitin-proteasome pathway, against preclinical models of multiple myeloma.* Blood, 2007. **110**(9): p. 3281-90.

101. Yang, H., et al., *Celastrol, a triterpene extracted from the Chinese "Thunder of God Vine," is a potent proteasome inhibitor and suppresses human prostate cancer growth in nude mice.* Cancer Res, 2006. **66**(9): p. 4758-65.

102. Sethi, G., et al., *Celastrol, a novel triterpene, potentiates TNF-induced apoptosis and suppresses invasion of tumor cells by inhibiting NF-kappaB-regulated gene products and TAK1-mediated NF-kappaB activation.* Blood, 2007. **109**(7): p. 2727-35.

103. Piva, R., et al., *CEP-18770: A novel, orally active proteasome inhibitor with a tumor-selective pharmacologic profile competitive with bortezomib.* Blood, 2008. **111**(5): p. 2765-75.

104. Kupperman, E., et al., *Evaluation of the proteasome inhibitor MLN9708 in preclinical models of human cancer.* Cancer Res, 2010. **70**(5): p. 1970-80.

105. Tsubuki, S., et al., *Differential inhibition of calpain and proteasome activities by peptidyl aldehydes of di-leucine and tri-leucine.* J Biochem, 1996. **119**(3): p. 572-6.

106. Raedler, L., *Velcade (Bortezomib) Receives 2 New FDA Indications: For Retreatment of Patients with Multiple Myeloma and for First-Line Treatment of Patients with Mantle-Cell Lymphoma.* Am Health Drug Benefits, 2015. **8**(Spec Feature): p. 135-40.

107. Vander Ark, A., J. Cao, and X. Li, *TGF-beta receptors: In and beyond TGF-beta signaling.* Cell Signal, 2018. **52**: p. 112-120.

108. Song, K., et al., *ERBB3, IGF1R, and TGFBR2 expression correlate with PDGFR expression in glioblastoma and participate in PDGFR inhibitor resistance of glioblastoma cells.* Am J Cancer Res, 2018. **8**(5): p. 792-809.

109. Jun, F., et al., *Epithelial membrane protein 3 regulates TGF-beta signaling activation in CD44-high glioblastoma.* Oncotarget, 2017. **8**(9): p. 14343-14358.

110. Kim, B.G., et al., *Novel therapies emerging in oncology to target the TGF-beta pathway.* J Hematol Oncol, 2021. **14**(1): p. 55.

111. Bueno, L., et al., *Semi-mechanistic modelling of the tumour growth inhibitory effects of LY2157299, a new type I receptor TGF-beta kinase antagonist, in mice.* Eur J Cancer, 2008. **44**(1): p. 142-50.

112. Son, J.Y., et al., *EW-7197, a novel ALK-5 kinase inhibitor, potently inhibits breast to lung metastasis.* Mol Cancer Ther, 2014. **13**(7): p. 1704-16.

113. Jin, C.H., et al., *Discovery of N-((4-([1,2,4]triazolo[1,5-a]pyridin-6-yl)-5-(6-methylpyridin-2-yl)-1H-imidazol-2 -yl)methyl)-2-fluoroaniline (EW-7197): a highly potent, selective, and orally bioavailable inhibitor of TGF-beta type I receptor kinase as cancer immunotherapeutic/antifibrotic agent.* J Med Chem, 2014. **57**(10): p. 4213-38.

114. Li, H.Y., et al., *Dihydropyrrolopyrazole transforming growth factor-beta type I receptor kinase domain inhibitors: a novel benzimidazole series with selectivity versus transforming growth factor-beta type II receptor kinase and mixed lineage kinase-7.* J Med Chem, 2006. **49**(6): p. 2138-42.

115. Liao, F., et al., *LSKL peptide alleviates subarachnoid fibrosis and hydrocephalus by inhibiting TSP1-mediated TGF-beta1 signaling activity following subarachnoid hemorrhage in rats.* Exp Ther Med, 2016. **12**(4): p. 2537-2543.

116. Wick, A., et al., *Phase 1b/2a study of galunisertib, a small molecule inhibitor of transforming growth factor-beta receptor I, in combination with standard temozolomide-based radiochemotherapy in patients with newly diagnosed malignant glioma.* Invest New Drugs, 2020. **38**(5): p. 1570-1579.

117. Fedele, M., E. Crescenzi, and L. Cerchia, *The POZ/BTB and AT-Hook Containing Zinc Finger 1 (PATZ1) Transcription Regulator: Physiological Functions and Disease Involvement.* Int J Mol Sci, 2017. **18**(12).

118. Quintero-Rivera, F., et al., *Disruption of a synaptotagmin (SYT14) associated with neurodevelopmental abnormalities.* Am J Med Genet A, 2007. **143A**(6): p. 558-63.

119. Richter, W.F., et al., *The Mediator complex as a master regulator of transcription by RNA polymerase II.* Nat Rev Mol Cell Biol, 2022.

120. Adler, D., et al., *MED15, encoding a subunit of the mediator complex, is overexpressed at high frequency in castration-resistant prostate cancer.* Int J Cancer, 2014. **135**(1): p. 19-26.

121. Klumper, N., et al., *Differential expression of Mediator complex subunit MED15 in testicular germ cell tumors.* Diagn Pathol, 2015. **10**: p. 165.

122. Offermann, A., et al., *MED15 overexpression in prostate cancer arises during androgen deprivation therapy via PI3K/mTOR signaling.* Oncotarget, 2017. **8**(5): p. 7964-7976.

123. Syring, I., et al., *The knockdown of the Mediator complex subunit MED15 restrains urothelial bladder cancer cells' malignancy.* Oncol Lett, 2018. **16**(3): p. 3013-3021.

124. White, D.N. and M.H.B. Stowell, *Room for Two: The Synaptophysin/Synaptobrevin Complex.* Front Synaptic Neurosci, 2021. **13**: p. 740318.

125. Chen, D.H., et al., *SYPL1 overexpression predicts poor prognosis of hepatocellular carcinoma and associates with epithelial-mesenchymal transition.* Oncol Rep, 2017. **38**(3): p. 1533-1542.

126. Yang, C. and Y. Wang, *Identification of differentiated functional modules in papillary thyroid carcinoma by analyzing differential networks.* J Cancer Res Ther, 2018. **14**(Supplement): p. S969-S974.

127. Pinzon Martin, S., P.H. Seeberger, and D. Varon Silva, *Mucins and Pathogenic Mucin-Like Molecules Are Immunomodulators During Infection and Targets for Diagnostics and Vaccines.* Front Chem, 2019. **7**: p. 710.

128. Sun, X., et al., *C1GALT1 in health and disease.* Oncol Lett, 2021. **22**(2): p. 589.

129. Lin, M.C., et al., *C1GALT1 predicts poor prognosis and is a potential therapeutic target in head and neck cancer.* Oncogene, 2018. **37**(43): p. 5780-5793.

130. Dong, X., et al., *A novel mechanism for C1GALT1 in the regulation of gastric cancer progression.* Cell Biosci, 2021. **11**(1): p. 166.

131. Grummt, I., *Life on a planet of its own: regulation of RNA polymerase I transcription in the nucleolus.* Genes Dev, 2003. **17**(14): p. 1691-702.

132. Drygin, D., W.G. Rice, and I. Grummt, *The RNA polymerase I transcription machinery: an emerging target for the treatment of cancer.* Annu Rev Pharmacol Toxicol, 2010. **50**: p. 131-56.

133. Rossetti, S., A.J. Wierzbicki, and N. Sacchi, *Mammary epithelial morphogenesis and early breast cancer. Evidence of involvement of basal components of the RNA Polymerase I transcription machinery.* Cell Cycle, 2016. **15**(18): p. 2515-26.

134. Ferreira, R., et al., *Targeting the RNA Polymerase I Transcription for Cancer Therapy Comes of Age.* Cells, 2020. **9**(2).

135. Peltonen, K., et al., *Identification of novel p53 pathway activating small-molecule compounds reveals unexpected similarities with known therapeutic agents.* PLoS One, 2010. **5**(9): p. e12996.

136. Drygin, D., et al., *Anticancer activity of CX-3543: a direct inhibitor of rRNA biogenesis.* Cancer Res, 2009. **69**(19): p. 7653-61.

137. Drygin, D., et al., *Targeting RNA polymerase I with an oral small molecule CX-5461 inhibits ribosomal RNA synthesis and solid tumor growth.* Cancer Res, 2011. **71**(4): p. 1418-30.

138. Hourcade, D., V.M. Holers, and J.P. Atkinson, *The regulators of complement activation (RCA) gene cluster.* Adv Immunol, 1989. **45**: p. 381-416.

139. Parente, R., et al., *Complement factor H in host defense and immune evasion.* Cell Mol Life Sci, 2017. **74**(9): p. 1605-1624.

140. Zhai, L., et al., *Tumor Cell IDO Enhances Immune Suppression and Decreases Survival Independent of Tryptophan Metabolism in Glioblastoma.* Clin Cancer Res, 2021. **27**(23): p. 6514-6528.

141. Bian, A., et al., *Circular RNA Complement Factor H (CFH) Promotes Glioma Progression by Sponging miR-149 and Regulating AKT1.* Med Sci Monit, 2018. **24**: p. 5704-5712.

142. Ajona, D., et al., *Expression of complement factor H by lung cancer cells: effects on the activation of the alternative pathway of complement.* Cancer Res, 2004. **64**(17): p. 6310-8.

143. Junnikkala, S., et al., *Secretion of soluble complement inhibitors factor H and factor H-like protein (FHL-1) by ovarian tumour cells.* Br J Cancer, 2002. **87**(10): p. 1119-27.

144. Jin, H.J., J. Kim, and J. Yu, *Androgen receptor genomic regulation.* Transl Androl Urol, 2013. **2**(3): p. 157-177.

145. Matsumoto, T., et al., *The androgen receptor in health and disease.* Annu Rev Physiol, 2013. **75**: p. 201-24.

146. Davey, R.A. and M. Grossmann, *Androgen Receptor Structure, Function and Biology: From Bench to Bedside.* Clin Biochem Rev, 2016. **37**(1): p. 3-15.

147. Hu, C., et al., *The androgen receptor expression and association with patient's survival in different cancers.* Genomics, 2020. **112**(2): p. 1926-1940.

148. Farina-Jeronimo, H., et al., *Androgen Receptor Activity Is Associated with Worse Survival in Glioblastoma.* J Integr Neurosci, 2022. **21**(3): p. 86.

149. Zalcman, N., et al., *Androgen receptor: a potential therapeutic target for glioblastoma.* Oncotarget, 2018. **9**(28): p. 19980-19993.

150. Lysiak, M., et al., *The sex-dependent role of the androgen receptor in glioblastoma: results of molecular analyses.* Mol Oncol, 2022.

151. Yang, J.D., et al., *Contribution of the Testosterone Androgen Receptor-PARD3B Signaling Axis to Tumorigenesis and Malignance of Glioblastoma Multiforme through Stimulating Cell Proliferation and Colony Formation.* J Clin Med, 2022. **11**(16).

152. Chen, T.C., et al., *AR ubiquitination induced by the curcumin analog suppresses growth of temozolomide-resistant glioblastoma through disrupting GPX4-Mediated redox homeostasis.* Redox Biol, 2020. **30**: p. 101413.

153. Rodriguez-Lozano, D.C., et al., *Testosterone Promotes Glioblastoma Cell Proliferation, Migration, and Invasion Through Androgen Receptor Activation.* Front Endocrinol (Lausanne), 2019. **10**: p. 16.

154. Rodriguez-Lozano, D.C., et al., *Dihydrotestosterone Induces Proliferation, Migration, and Invasion of Human Glioblastoma Cell Lines.* Onco Targets Ther, 2020. **13**: p. 8813-8823.

155. Yu, X., et al., *Androgen receptor signaling regulates growth of glioblastoma multiforme in men.* Tumour Biol, 2015. **36**(2): p. 967-72.

156. Werner, C.K., et al., *Expression of the Androgen Receptor Governs Radiation Resistance in a Subset of Glioblastomas Vulnerable to Antiandrogen Therapy.* Mol Cancer Ther, 2020. **19**(10): p. 2163-2174.

157. Zhao, N., et al., *Androgen Receptor, Although Not a Specific Marker For, Is a Novel Target to Suppress Glioma Stem Cells as a Therapeutic Strategy for Glioblastoma.* Front Oncol, 2021. **11**: p. 616625.

158. Orozco, M., et al., *Dutasteride combined with androgen receptor antagonists inhibit glioblastoma U87 cell metabolism, proliferation, and invasion capacity: Androgen regulation.* Steroids, 2020. **164**: p. 108733.

159. Kim, T.J., Y.H. Lee, and K.C. Koo, *Current Status and Future Perspectives of Androgen Receptor Inhibition Therapy for Prostate Cancer: A Comprehensive Review.* Biomolecules, 2021. **11**(4).

160. Kono, M., et al., *Androgen Receptor Function and Androgen Receptor-Targeted Therapies in Breast Cancer: A Review.* JAMA Oncol, 2017. **3**(9): p. 1266-1273.

161. Chen, Y., et al., *Second generation androgen receptor antagonists and challenges in prostate cancer treatment.* Cell Death Dis, 2022. **13**(7): p. 632.

162. Clegg, N.J., et al., *ARN-509: a novel antiandrogen for prostate cancer treatment.* Cancer Res, 2012. **72**(6): p. 1494-503.

163. Furr, B.J., et al., *ICI 176,334: a novel non-steroidal, peripherally selective antiandrogen.* J Endocrinol, 1987. **113**(3): p. R7-9.

164. Furr, B.J., *ICI 176,334: a novel non-steroidal, peripherally-selective antiandrogen.* Prog Clin Biol Res, 1988. **260**: p. 13-26.

165. Moilanen, A.M., et al., *Discovery of ODM-201, a new-generation androgen receptor inhibitor targeting resistance mechanisms to androgen signaling-directed prostate cancer therapies.* Sci Rep, 2015. **5**: p. 12007.

166. Tran, C., et al., *Development of a second-generation antiandrogen for treatment of advanced prostate cancer.* Science, 2009. **324**(5928): p. 787-90.

167. Katchen, B. and S. Buxbaum, *Disposition of a new, nonsteroid, antiandrogen, alpha,alpha,alpha-trifluoro-2-methyl-4'-nitro-m-propionotoluidide (Flutamide), in men following a single oral 200 mg dose.* J Clin Endocrinol Metab, 1975. **41**(2): p. 373-9.

168. Harris, M.G., et al., *Nilutamide. A review of its pharmacodynamic and pharmacokinetic properties, and therapeutic efficacy in prostate cancer.* Drugs Aging, 1993. **3**(1): p. 9-25.

169. Belanger, A., A. Dupont, and F. Labrie, *Inhibition of basal and adrenocorticotropin-stimulated plasma levels of adrenal androgens after treatment with an antiandrogen in castrated patients with prostatic cancer.* J Clin Endocrinol Metab, 1984. **59**(3): p. 422-6.

170. Wollman, A.L. and J.B. Hamilton, *Prevention by cyproterone acetate of androgenic, but not of gonadotrophic, elicitation of persistent estrus in rats.* Endocrinology, 1967. **81**(2): p. 350-6.

171. Brave, M., et al., *An FDA Review of Drug Development in Nonmetastatic Castration-resistant Prostate Cancer.* Clin Cancer Res, 2020. **26**(18): p. 4717-4722.

172. Kanai, T., et al., *Identification of STAT5A and STAT5B target genes in human T cells.* PLoS One, 2014. **9**(1): p. e86790.

173. Kanai, T., J. Jenks, and K.C. Nadeau, *The STAT5b Pathway Defect and Autoimmunity.* Front Immunol, 2012. **3**: p. 234.

174. Dubois, N., et al., *STAT5b is a marker of poor prognosis, rather than a therapeutic target in glioblastomas.* Int J Oncol, 2022. **61**(4).

175. Roos, A., et al., *EGFRvIII-Stat5 Signaling Enhances Glioblastoma Cell Migration and Survival.* Mol Cancer Res, 2018. **16**(7): p. 1185-1195.

176. Latha, K., et al., *Nuclear EGFRvIII-STAT5b complex contributes to glioblastoma cell survival by direct activation of the Bcl-XL promoter.* Int J Cancer, 2013. **132**(3): p. 509-20.

177. Moyama, C., et al., *Stat5b inhibition blocks proliferation and tumorigenicity of glioblastoma stem cells derived from a de novo murine brain cancer model.* Am J Cancer Res, 2022. **12**(3): p. 1129-1142.

178. Liang, Q.C., et al., *Inhibition of transcription factor STAT5b suppresses proliferation, induces G1 cell cycle arrest and reduces tumor cell invasion in human glioblastoma multiforme cells.* Cancer Lett, 2009. **273**(1): p. 164-71.

179. Loh, C.Y., et al., *Signal Transducer and Activator of Transcription (STATs) Proteins in Cancer and Inflammation: Functions and Therapeutic Implication.* Front Oncol, 2019. **9**: p. 48.

180. Muller, J., et al., *Discovery of chromone-based inhibitors of the transcription factor STAT5.* Chembiochem, 2008. **9**(5): p. 723-7.

181. Chien, C.M., et al., *Novel indoloquinoline derivative, IQDMA, suppresses STAT5 phosphorylation and induces apoptosis in HL-60 cells.* Chem Biol Interact, 2008. **176**(1): p. 40-7.

182. Nelson, E.A., et al., *The STAT5 inhibitor pimozide decreases survival of chronic myelogenous leukemia cells resistant to kinase inhibitors.* Blood, 2011. **117**(12): p. 3421-9.

183. Wingelhofer, B., et al., *Pharmacologic inhibition of STAT5 in acute myeloid leukemia.* Leukemia, 2018. **32**(5): p. 1135-1146.

184. Maranto, C., et al., *Prospects for Clinical Development of Stat5 Inhibitor IST5-002: High Transcriptomic Specificity in Prostate Cancer and Low Toxicity In Vivo.* Cancers (Basel), 2020. **12**(11).

185. Liu, Y., et al., *BD750, a benzothiazole derivative, inhibits T cell proliferation by affecting the JAK3/STAT5 signalling pathway.* Br J Pharmacol, 2013. **168**(3): p. 632-43.

186. Kruger, N.J. and A. von Schaewen, *The oxidative pentose phosphate pathway: structure and organisation.* Curr Opin Plant Biol, 2003. **6**(3): p. 236-46.

187. Luzzatto, L., M. Ally, and R. Notaro, *Glucose-6-phosphate dehydrogenase deficiency.* Blood, 2020. **136**(11): p. 1225-1240.

188. Song, J., et al., *The Multiple Roles of Glucose-6-Phosphate Dehydrogenase in Tumorigenesis and Cancer Chemoresistance.* Life (Basel), 2022. **12**(2).

189. Xu, X., et al., *Rewiring of purine metabolism in response to acidosis stress in glioma stem cells.* Cell Death Dis, 2021. **12**(3): p. 277.

190. Stanke, K.M., C. Wilson, and S. Kidambi, *High Expression of Glycolytic Genes in Clinical Glioblastoma Patients Correlates With Lower Survival.* Front Mol Biosci, 2021. **8**: p. 752404.

191. Yang, C.A., et al., *G6PD as a predictive marker for glioma risk, prognosis and chemosensitivity.* J Neurooncol, 2018. **139**(3): p. 661-670.

192. Sun, M., et al., *PIKE-A promotes glioblastoma growth by driving PPP flux through increasing G6PD expression mediated by phosphorylation of STAT3.* Biochem Pharmacol, 2021. **192**: p. 114736.

193. Carmona, A. and R.A. Freedland, *Effect of 6-aminonicotinamide on pentose cycle activity in isolated hepatocytes.* Int J Biochem, 1990. **22**(6): p. 595-9.

194. Scicinski, J., et al., *Preclinical evaluation of the metabolism and disposition of RRx-001, a novel investigative anticancer agent.* Drug Metab Dispos, 2012. **40**(9): p. 1810-6.

195. Das, D.S., et al., *A novel hypoxia-selective epigenetic agent RRx-001 triggers apoptosis and overcomes drug resistance in multiple myeloma cells.* Leukemia, 2016. **30**(11): p. 2187-2197.

196. Oronsky, B., T. Reid, and P. Cabrales, *Vascular priming with RRx-001 to increase the uptake and accumulation of temozolomide and irinotecan in orthotopically implanted gliomas.* J Drug Target, 2021. **29**(9): p. 998-1003.

197. Oronsky, B., et al., *Discovery of RRx-001, a Myc and CD47 Downregulating Small Molecule with Tumor Targeted Cytotoxicity and Healthy Tissue Cytoprotective Properties in Clinical Development.* J Med Chem, 2021. **64**(11): p. 7261-7271.

198. Mele, L., et al., *A new inhibitor of glucose-6-phosphate dehydrogenase blocks pentose phosphate pathway and suppresses malignant proliferation and metastasis in vivo.* Cell Death Dis, 2018. **9**(5): p. 572.

199. Ghergurovich, J.M., et al., *A small molecule G6PD inhibitor reveals immune dependence on pentose phosphate pathway.* Nat Chem Biol, 2020. **16**(7): p. 731-739.

200. Luo, Z., et al., *Discovery and characterization of a novel glucose-6-phosphate dehydrogenase (G6PD) inhibitor via high-throughput screening.* Bioorg Med Chem Lett, 2021. **40**: p. 127905.

201. Lu, S.C., *Glutathione synthesis.* Biochim Biophys Acta, 2013. **1830**(5): p. 3143-53.

202. Griffith, O.W., *Biologic and pharmacologic regulation of mammalian glutathione synthesis.* Free Radic Biol Med, 1999. **27**(9-10): p. 922-35.

203. Bu, X., et al., *CD147 confers temozolomide resistance of glioma cells via the regulation of beta-TrCP/Nrf2 pathway.* Int J Biol Sci, 2021. **17**(12): p. 3013-3023.

204. Landras, A., et al., *CD147 Is a Promising Target of Tumor Progression and a Prognostic Biomarker.* Cancers (Basel), 2019. **11**(11).

205. Trautwein, C., et al., *Tissue metabolites in diffuse glioma and their modulations by IDH1 mutation, histology, and treatment.* JCI Insight, 2022. **7**(3).

206. Fack, F., et al., *Altered metabolic landscape in IDH-mutant gliomas affects phospholipid, energy, and oxidative stress pathways.* EMBO Mol Med, 2017. **9**(12): p. 1681-1695.

207. Griffith, O.W. and A. Meister, *Potent and specific inhibition of glutathione synthesis by buthionine sulfoximine (S-n-butyl homocysteine sulfoximine).* J Biol Chem, 1979. **254**(16): p. 7558-60.

208. Backos, D.S., et al., *Glycation of glutamate cysteine ligase by 2-deoxy-d-ribose and its potential impact on chemoresistance in glioblastoma.* Neurochem Res, 2013. **38**(9): p. 1838-49.

209. Ran, S., et al., *NaAsO2 decreases GSH synthesis by inhibiting GCLC and induces apoptosis through Hela cell mitochondrial damage, mediating the activation of the NF-kappaB/miR-21 signaling pathway.* Ecotoxicol Environ Saf, 2022. **234**: p. 113380.

210. Villablanca, J.G., et al., *A Phase I New Approaches to Neuroblastoma Therapy Study of Buthionine Sulfoximine and Melphalan With Autologous Stem Cells for Recurrent/Refractory High-Risk Neuroblastoma.* Pediatr Blood Cancer, 2016. **63**(8): p. 1349-56.

211. Anderson, C.P., et al., *Pilot study of intravenous melphalan combined with continuous infusion L-S,R-buthionine sulfoximine for children with recurrent neuroblastoma.* Pediatr Blood Cancer, 2015. **62**(10): p. 1739-46.

212. Lee, S., S.M. Kim, and R.T. Lee, *Thioredoxin and thioredoxin target proteins: from molecular mechanisms to functional significance.* Antioxid Redox Signal, 2013. **18**(10): p. 1165-207.

213. Mohammadi, F., et al., *The thioredoxin system and cancer therapy: a review.* Cancer Chemother Pharmacol, 2019. **84**(5): p. 925-935.

214. Erdi, F., et al., *New Clues in the Malignant Progression of Glioblastoma: Can the Thioredoxin System Play a Role?* Turk Neurosurg, 2018. **28**(1): p. 7-12.

215. Yao, A., et al., *Thioredoxin System Protein Expression Is Associated with Poor Clinical Outcome in Adult and Paediatric Gliomas and Medulloblastomas.* Mol Neurobiol, 2020. **57**(7): p. 2889-2901.

216. Kaya, B., et al., *Intratumoral hemorrhage-related differences in the expression of vascular endothelial growth factor, basic fibroblast growth factor and thioredoxin reductase 1 in human glioblastoma.* Mol Clin Oncol, 2016. **5**(4): p. 343-346.

217. Branco, V., et al., *Thioredoxin, Glutathione and Related Molecules in Tumors of the Nervous System.* Curr Med Chem, 2020. **27**(12): p. 1878-1900.

218. Jovanovic, M., et al., *Novel TrxR1 Inhibitors Show Potential for Glioma Treatment by Suppressing the Invasion and Sensitizing Glioma Cells to Chemotherapy.* Front Mol Biosci, 2020. **7**: p. 586146.

219. Zhang, Y., et al., *TIGAR knockdown radiosensitizes TrxR1-overexpressing glioma in vitro and in vivo via inhibiting Trx1 nuclear transport.* Sci Rep, 2017. **7**: p. 42928.

220. Wang, H., et al., *Auranofin radiosensitizes tumor cells through targeting thioredoxin reductase and resulting overproduction of reactive oxygen species.* Oncotarget, 2017. **8**(22): p. 35728-35742.

221. Patwardhan, R.S., D. Sharma, and S.K. Sandur, *Thioredoxin reductase: An emerging pharmacologic target for radiosensitization of cancer.* Transl Oncol, 2022. **17**: p. 101341.

222. Yao, A., et al., *Cytotoxic and Radiosensitising Effects of a Novel Thioredoxin Reductase Inhibitor in Brain Cancers.* Mol Neurobiol, 2022. **59**(6): p. 3546-3563.

223. Sutton, B.M., et al., *Oral gold. Antiarthritic properties of alkylphosphinegold coordination complexes.* J Med Chem, 1972. **15**(11): p. 1095-8.

224. Rigobello, M.P., et al., *Effect of auranofin on the mitochondrial generation of hydrogen peroxide. Role of thioredoxin reductase.* Free Radic Res, 2005. **39**(7): p. 687-95.

225. Gandin, V., et al., *Cancer cell death induced by phosphine gold(I) compounds targeting thioredoxin reductase.* Biochem Pharmacol, 2010. **79**(2): p. 90-101.

226. Zhang, X., et al., *Repurposing of auranofin: Thioredoxin reductase remains a primary target of the drug.* Biochimie, 2019. **162**: p. 46-54.

227. Javvadi, P., et al., *Thioredoxin reductase-1 mediates curcumin-induced radiosensitization of squamous carcinoma cells.* Cancer Res, 2010. **70**(5): p. 1941-50.

228. Kalin, S.N., A. Altay, and H. Budak, *Diffractaic acid, a novel TrxR1 inhibitor, induces cytotoxicity, apoptosis, and antimigration in human breast cancer cells.* Chem Biol Interact, 2022. **361**: p. 109984.

229. Wang, L., et al., *Ethaselen: a potent mammalian thioredoxin reductase 1 inhibitor and novel organoselenium anticancer agent.* Free Radic Biol Med, 2012. **52**(5): p. 898-908.

230. Rice, K.P., et al., *Thioredoxin reductase is inhibited by the carbamoylating activity of the anticancer sulfonylhydrazine drug laromustine.* Mol Cell Biochem, 2012. **370**(1-2): p. 199-207.

231. Lu, J., et al., *Inhibition of Mammalian thioredoxin reductase by some flavonoids: implications for myricetin and quercetin anticancer activity.* Cancer Res, 2006. **66**(8): p. 4410-8.

232. Zhang, Q., et al., *Piperlongumine, a Novel TrxR1 Inhibitor, Induces Apoptosis in Hepatocellular Carcinoma Cells by ROS-Mediated ER Stress.* Front Pharmacol, 2019. **10**: p. 1180.

233. Zhang, J., et al., *Inhibition of Thioredoxin Reductase by Santamarine Conferring Anticancer Effect in HeLa Cells.* Front Mol Biosci, 2021. **8**: p. 710676.

234. Stafford, W.C., et al., *Irreversible inhibition of cytosolic thioredoxin reductase 1 as a mechanistic basis for anticancer therapy.* Sci Transl Med, 2018. **10**(428).

235. Dos Santos, D.C., et al., *IP-Se-06, a Selenylated Imidazo[1,2-a]pyridine, Modulates Intracellular Redox State and Causes Akt/mTOR/HIF-1alpha and MAPK Signaling Inhibition, Promoting Antiproliferative Effect and Apoptosis in Glioblastoma Cells.* Oxid Med Cell Longev, 2022. **2022**: p. 3710449.

236. Arner, E.S. and A. Holmgren, *The thioredoxin system in cancer.* Semin Cancer Biol, 2006. **16**(6): p. 420-6.

237. Karlenius, T.C. and K.F. Tonissen, *Thioredoxin and Cancer: A Role for Thioredoxin in all States of Tumor Oxygenation.* Cancers (Basel), 2010. **2**(2): p. 209-32.

238. Tian, Y., et al., *Diallyl trisulfide sensitizes radiation therapy on glioblastoma through directly targeting thioredoxin 1.* Free Radic Biol Med, 2022. **189**: p. 157-168.

239. Lu, J., E.H. Chew, and A. Holmgren, *Targeting thioredoxin reductase is a basis for cancer therapy by arsenic trioxide.* Proc Natl Acad Sci U S A, 2007. **104**(30): p. 12288-93.

240. Welsh, S.J., et al., *The thioredoxin redox inhibitors 1-methylpropyl 2-imidazolyl disulfide and pleurotin inhibit hypoxia-induced factor 1alpha and vascular endothelial growth factor formation.* Mol Cancer Ther, 2003. **2**(3): p. 235-43.

241. Ramanathan, R.K., et al., *A randomized phase II study of PX-12, an inhibitor of thioredoxin in patients with advanced cancer of the pancreas following progression after a gemcitabine-containing combination.* Cancer Chemother Pharmacol, 2011. **67**(3): p. 503-9.

242. Ramanathan, R.K., et al., *A phase I trial of PX-12, a small-molecule inhibitor of thioredoxin-1, administered as a 72-hour infusion every 21 days in patients with advanced cancers refractory to standard therapy.* Invest New Drugs, 2012. **30**(4): p. 1591-6.

243. Baker, A.F., et al., *A phase IB trial of 24-hour intravenous PX-12, a thioredoxin-1 inhibitor, in patients with advanced gastrointestinal cancers.* Invest New Drugs, 2013. **31**(3): p. 631-641.

244. Padavannil, A., et al., *The Mysterious Multitude: Structural Perspective on the Accessory Subunits of Respiratory Complex I.* Front Mol Biosci, 2021. **8**: p. 798353.

245. Pan, Y.B., et al., *Transcriptome analyses reveal molecular mechanisms underlying phenotypic differences among transcriptional subtypes of glioblastoma.* J Cell Mol Med, 2020. **24**(7): p. 3901-3916.

246. Bachvarov, D., et al., *Gene expression patterns of chemoresistant and chemosensitive serous epithelial ovarian tumors with possible predictive value in response to initial chemotherapy.* Int J Oncol, 2006. **29**(4): p. 919-33.

247. Chang, Z., X. Miao, and W. Zhao, *Identification of Prognostic Dosage-Sensitive Genes in Colorectal Cancer Based on Multi-Omics.* Front Genet, 2019. **10**: p. 1310.

248. Sotgia, F. and M.P. Lisanti, *Mitochondrial biomarkers predict tumor progression and poor overall survival in gastric cancers: Companion diagnostics for personalized medicine.* Oncotarget, 2017. **8**(40): p. 67117-67128.

249. Sterne, J., *[Treatment of diabetes mellitus with N,N-dimethylguanylguanidine (LA. 6023, glucophage)].* Therapie, 1959. **14**: p. 625-30.

250. El-Mir, M.Y., et al., *Dimethylbiguanide inhibits cell respiration via an indirect effect targeted on the respiratory chain complex I.* J Biol Chem, 2000. **275**(1): p. 223-8.

251. OWEN, M.R., E. DORAN, and A.P. HALESTRAP, *Evidence that metformin exerts its anti-diabetic effects through inhibition of complex 1 of the mitochondrial respiratory chain.* Biochemical Journal, 2000. **348**(3): p. 607-614.

252. Fontaine, E., *Metformin-Induced Mitochondrial Complex I Inhibition: Facts, Uncertainties, and Consequences.* Front Endocrinol (Lausanne), 2018. **9**: p. 753.

253. Kawada, M., et al., *Intervenolin, a new antitumor compound with anti-Helicobacter pylori activity, from Nocardia sp. ML96-86F2.* J Antibiot (Tokyo), 2013. **66**(9): p. 543-8.

254. Yoshida, J., et al., *Mitochondrial complex I inhibitors suppress tumor growth through concomitant acidification of the intra- and extracellular environment.* iScience, 2021. **24**(12): p. 103497.

255. Shi, Y., et al., *Gboxin is an oxidative phosphorylation inhibitor that targets glioblastoma.* Nature, 2019. **567**(7748): p. 341-346.

256. Zampieri, L.X., et al., *Olaparib Is a Mitochondrial Complex I Inhibitor That Kills Temozolomide-Resistant Human Glioblastoma Cells.* Int J Mol Sci, 2021. **22**(21).

257. Luna Yolba, R., et al., *EVT-701 is a novel selective and safe mitochondrial complex 1 inhibitor with potent anti-tumor activity in models of solid cancers.* Pharmacol Res Perspect, 2021. **9**(5): p. e00854.

258. Pant, S., et al., *A first-in-human dose-escalation study of ME-143, a second generation NADH oxidase inhibitor, in patients with advanced solid tumors.* Invest New Drugs, 2014. **32**(1): p. 87-93.

259. Lim, S.C., K.T. Carey, and M. McKenzie, *Anti-cancer analogues ME-143 and ME-344 exert toxicity by directly inhibiting mitochondrial NADH: ubiquinone oxidoreductase (Complex I).* Am J Cancer Res, 2015. **5**(2): p. 689-701.

260. Bendell, J.C., et al., *Phase 1, open-label, dose escalation, safety, and pharmacokinetics study of ME-344 as a single agent in patients with refractory solid tumors.* Cancer, 2015. **121**(7): p. 1056-63.

261. Molina, J.R., et al., *An inhibitor of oxidative phosphorylation exploits cancer vulnerability.* Nat Med, 2018. **24**(7): p. 1036-1046.

262. Helbig, L., et al., *BAY 87-2243, a novel inhibitor of hypoxia-induced gene activation, improves local tumor control after fractionated irradiation in a schedule-dependent manner in head and neck human xenografts.* Radiat Oncol, 2014. **9**: p. 207.

263. Schockel, L., et al., *Targeting mitochondrial complex I using BAY 87-2243 reduces melanoma tumor growth.* Cancer Metab, 2015. **3**: p. 11.

264. Degli Esposti, M., *Inhibitors of NADH-ubiquinone reductase: an overview.* Biochim Biophys Acta, 1998. **1364**(2): p. 222-35.

265. Ashton, T.M., et al., *Oxidative Phosphorylation as an Emerging Target in Cancer Therapy.* Clin Cancer Res, 2018. **24**(11): p. 2482-2490.

266. Pombo, C.M., et al., *MST Kinases and Metabolism.* Endocrinology, 2019. **160**(5): p. 1111-1118.

267. Mahlapuu, M., et al., *GCKIII kinases in lipotoxicity: Roles in NAFLD and beyond.* Hepatol Commun, 2022.

268. Lim, S., et al., *Identification of the kinase STK25 as an upstream activator of LATS signaling.* Nat Commun, 2019. **10**(1): p. 1547.

269. Wu, F., et al., *STK25-induced inhibition of aerobic glycolysis via GOLPH3-mTOR pathway suppresses cell proliferation in colorectal cancer.* J Exp Clin Cancer Res, 2018. **37**(1): p. 144.

270. Zhang, Y., et al., *STK25 enhances hepatocellular carcinoma progression through the STRN/AMPK/ACC1 pathway.* Cancer Cell Int, 2022. **22**(1): p. 4.

271. Kurhe, Y., et al., *Antagonizing STK25 Signaling Suppresses the Development of Hepatocellular Carcinoma Through Targeting Metabolic, Inflammatory, and Pro-Oncogenic Pathways.* Cell Mol Gastroenterol Hepatol, 2022. **13**(2): p. 405-423.

272. Zhu, Z., et al., *Glutathione reductase mediates drug resistance in glioblastoma cells by regulating redox homeostasis.* J Neurochem, 2018. **144**(1): p. 93-104.

273. McLoughlin, M.R., et al., *TrxR1, Gsr, and oxidative stress determine hepatocellular carcinoma malignancy.* Proc Natl Acad Sci U S A, 2019. **116**(23): p. 11408-11417.

274. Baity, M., et al., *Glutathione reductase (GSR) gene deletion and chromosome 8 aneuploidy in primary lung cancers detected by fluorescence in situ hybridization.* Am J Cancer Res, 2019. **9**(6): p. 1201-1211.

275. Gopi, L.K. and B.L. Kidder, *Integrative pan cancer analysis reveals epigenomic variation in cancer type and cell specific chromatin domains.* Nat Commun, 2021. **12**(1): p. 1419.

276. Chung, P.M., R.E. Cappel, and H.F. Gilbert, *Inhibition of glutathione disulfide reductase by glutathione.* Arch Biochem Biophys, 1991. **288**(1): p. 48-53.

277. Seefeldt, T., et al., *Characterization of a novel dithiocarbamate glutathione reductase inhibitor and its use as a tool to modulate intracellular glutathione.* J Biol Chem, 2009. **284**(5): p. 2729-2737.

278. Johnson, F.D., et al., *Characterization of a small molecule inhibitor of disulfide reductases that induces oxidative stress and lethality in lung cancer cells.* Cell Rep, 2022. **38**(6): p. 110343.

279. Cole, S.P., *Targeting multidrug resistance protein 1 (MRP1, ABCC1): past, present, and future.* Annu Rev Pharmacol Toxicol, 2014. **54**: p. 95-117.

280. Dean, M., K. Moitra, and R. Allikmets, *The human ATP-binding cassette (ABC) transporter superfamily.* Hum Mutat, 2022. **43**(9): p. 1162-1182.

281. Kunicka, T. and P. Soucek, *Importance of ABCC1 for cancer therapy and prognosis.* Drug Metab Rev, 2014. **46**(3): p. 325-42.

282. Xiao, H., et al., *Clinically-Relevant ABC Transporter for Anti-Cancer Drug Resistance.* Front Pharmacol, 2021. **12**: p. 648407.

283. Li, Y. and X. Gao, *LINC00883 Promotes Drug Resistance of Glioma Through a microRNA-136/NEK1-Dependent Mechanism.* Front Oncol, 2021. **11**: p. 692265.

284. Mahinfar, P., et al., *The Role of microRNAs in Multidrug Resistance of Glioblastoma.* Cancers (Basel), 2022. **14**(13).

285. Wu, C., et al., *LINC00470 promotes tumour proliferation and invasion, and attenuates chemosensitivity through the LINC00470/miR-134/Myc/ABCC1 axis in glioma.* J Cell Mol Med, 2020. **24**(20): p. 12094-12106.

286. Li, Y., et al., *miR-1268a regulates ABCC1 expression to mediate temozolomide resistance in glioblastoma.* J Neurooncol, 2018. **138**(3): p. 499-508.

287. Chen, X.R., Y.G. Zhang, and Q. Wang, *miR-9-5p Mediates ABCC1 to Elevate the Sensitivity of Glioma Cells to Temozolomide.* Front Oncol, 2021. **11**: p. 661653.

288. Jones, T.R., et al., *Pharmacology of L-660,711 (MK-571): a novel potent and selective leukotriene D4 receptor antagonist.* Can J Physiol Pharmacol, 1989. **67**(1): p. 17-28.

289. Burkhart, C.A., et al., *Small-molecule multidrug resistance-associated protein 1 inhibitor reversan increases the therapeutic index of chemotherapy in mouse models of neuroblastoma.* Cancer Res, 2009. **69**(16): p. 6573-80.

290. Silbermann, K., et al., *Identification of Thienopyrimidine Scaffold as an Inhibitor of the ABC Transport Protein ABCC1 (MRP1) and Related Transporters Using a Combined Virtual Screening Approach.* J Med Chem, 2019. **62**(9): p. 4383-4400.

291. Hirano, Y., et al., *A heterodimeric complex that promotes the assembly of mammalian 20S proteasomes.* Nature, 2005. **437**(7063): p. 1381-5.

292. Voutsadakis, I.A., *Proteasome expression and activity in cancer and cancer stem cells.* Tumour Biol, 2017. **39**(3): p. 1010428317692248.

293. Arlt, A., et al., *Increased proteasome subunit protein expression and proteasome activity in colon cancer relate to an enhanced activation of nuclear factor E2-related factor 2 (Nrf2).* Oncogene, 2009. **28**(45): p. 3983-96.

294. Lee, D., et al., *miR-484 is associated with disease recurrence and promotes migration in prostate cancer.* Biosci Rep, 2020. **40**(5).

295. Li, K. and T. Liu, *Evaluation of Oncogene NUP37 as a Potential Novel Biomarker in Breast Cancer.* Front Oncol, 2021. **11**: p. 669655.

296. Alshabi, A.M., et al., *Identification of Crucial Candidate Genes and Pathways in Glioblastoma Multiform by Bioinformatics Analysis.* Biomolecules, 2019. **9**(5).

297. Tanaka, K., *The proteasome: overview of structure and functions.* Proc Jpn Acad Ser B Phys Biol Sci, 2009. **85**(1): p. 12-36.

298. Yongjun Zhang, M.M., et al., *Association between TGM5, PPAP2B and PSMA4 polymorphisms and NSCLC in never-smoking Chinese population.* J Cancer Res Ther, 2013. **9**(4): p. 660-3.

299. Guo, M., et al., *The Study on the Clinical Phenotype and Function of HPRT1 Gene.* Child Neurol Open, 2022. **9**: p. 2329048X221108821.

300. Townsend, M.H., R.A. Robison, and K.L. O'Neill, *A review of HPRT and its emerging role in cancer.* Med Oncol, 2018. **35**(6): p. 89.

301. M, J.S., et al., *Hypoxanthine Phosphoribosyl Transferase 1 Is Upregulated, Predicts Clinical Outcome and Controls Gene Expression in Breast Cancer.* Cancers (Basel), 2020. **12**(6).

302. Ahmadi, M., et al., *Overexpression of HPRT1 is associated with poor prognosis in head and neck squamous cell carcinoma.* FEBS Open Bio, 2021. **11**(9): p. 2525-2540.

303. Wu, T., et al., *HPRT1 Promotes Chemoresistance in Oral Squamous Cell Carcinoma via Activating MMP1/PI3K/Akt Signaling Pathway.* Cancers (Basel), 2022. **14**(4).

304. Lane, A.N. and T.W. Fan, *Regulation of mammalian nucleotide metabolism and biosynthesis.* Nucleic Acids Res, 2015. **43**(4): p. 2466-85.

305. Sun, H., et al., *RRM2 is a potential prognostic biomarker with functional significance in glioma.* Int J Biol Sci, 2019. **15**(3): p. 533-543.

306. Rasmussen, R.D., et al., *BRCA1-regulated RRM2 expression protects glioblastoma cells from endogenous replication stress and promotes tumorigenicity.* Nat Commun, 2016. **7**: p. 13398.

307. Jiang, H., et al., *RRM2 Mediates the Anti-Tumor Effect of the Natural Product Pectolinarigenin on Glioblastoma Through Promoting CDK1 Protein Degradation by Increasing Autophagic Flux.* Front Oncol, 2022. **12**: p. 887294.

308. Li, C., et al., *RRM2 promotes the progression of human glioblastoma.* J Cell Physiol, 2018. **233**(10): p. 6759-6767.

309. Li, J., et al., *Suppression of RRM2 inhibits cell proliferation, causes cell cycle arrest and promotes the apoptosis of human neuroblastoma cells and in human neuroblastoma RRM2 is suppressed following chemotherapy.* Oncol Rep, 2018. **40**(1): p. 355-360.

310. Shi, S.C., Y. Zhang, and T. Wang, *High RRM2 expression has poor prognosis in specific types of breast cancer.* PLoS One, 2022. **17**(3): p. e0265195.

311. Zhuang, S., et al., *RRM2 elicits the metastatic potential of breast cancer cells by regulating cell invasion, migration and VEGF expression via the PI3K/AKT signaling.* Oncol Lett, 2020. **19**(4): p. 3349-3355.

312. Zhan, Y., et al., *Inhibiting RRM2 to enhance the anticancer activity of chemotherapy.* Biomed Pharmacother, 2021. **133**: p. 110996.

313. Jiang, X., et al., *RRM2 silencing suppresses malignant phenotype and enhances radiosensitivity via activating cGAS/STING signaling pathway in lung adenocarcinoma.* Cell Biosci, 2021. **11**(1): p. 74.

314. Abdel-Rahman, M.A., M. Mahfouz, and H.O. Habashy, *RRM2 expression in different molecular subtypes of breast cancer and its prognostic significance.* Diagn Pathol, 2022. **17**(1): p. 1.

315. Ohmura, S., et al., *Translational evidence for RRM2 as a prognostic biomarker and therapeutic target in Ewing sarcoma.* Mol Cancer, 2021. **20**(1): p. 97.

316. Zhou, B., et al., *A small-molecule blocking ribonucleotide reductase holoenzyme formation inhibits cancer cell growth and overcomes drug resistance.* Cancer Res, 2013. **73**(21): p. 6484-93.

317. Liu, X., et al., *Inhibition of hepatitis B virus replication by targeting ribonucleotide reductase M2 protein.* Biochem Pharmacol, 2016. **103**: p. 118-28.

318. Xie, Y., et al., *Preclinical validation and phase I trial of 4-hydroxysalicylanilide, targeting ribonucleotide reductase mediated dNTP synthesis in multiple myeloma.* J Biomed Sci, 2022. **29**(1): p. 32.

319. Koppula, P., L. Zhuang, and B. Gan, *Cystine transporter SLC7A11/xCT in cancer: ferroptosis, nutrient dependency, and cancer therapy.* Protein Cell, 2021. **12**(8): p. 599-620.

320. Koppula, P., et al., *Amino acid transporter SLC7A11/xCT at the crossroads of regulating redox homeostasis and nutrient dependency of cancer.* Cancer Commun (Lond), 2018. **38**(1): p. 12.

321. Liu, J., X. Xia, and P. Huang, *xCT: A Critical Molecule That Links Cancer Metabolism to Redox Signaling.* Mol Ther, 2020. **28**(11): p. 2358-2366.

322. Robert, S.M., et al., *SLC7A11 expression is associated with seizures and predicts poor survival in patients with malignant glioma.* Sci Transl Med, 2015. **7**(289): p. 289ra86.

323. Polewski, M.D., et al., *SLC7A11 Overexpression in Glioblastoma Is Associated with Increased Cancer Stem Cell-Like Properties.* Stem Cells Dev, 2017. **26**(17): p. 1236-1246.

324. Jyotsana, N., K.T. Ta, and K.E. DelGiorno, *The Role of Cystine/Glutamate Antiporter SLC7A11/xCT in the Pathophysiology of Cancer.* Front Oncol, 2022. **12**: p. 858462.

325. Dixon, S.J., et al., *Pharmacological inhibition of cystine-glutamate exchange induces endoplasmic reticulum stress and ferroptosis.* Elife, 2014. **3**: p. e02523.

326. Jin, C., et al., *Inhibition of SLC7A11 by Sulfasalazine Enhances Osteogenic Differentiation of Mesenchymal Stem Cells by Modulating BMP2/4 Expression and Suppresses Bone Loss in Ovariectomized Mice.* J Bone Miner Res, 2017. **32**(3): p. 508-521.

327. Zhang, Y., et al., *Imidazole Ketone Erastin Induces Ferroptosis and Slows Tumor Growth in a Mouse Lymphoma Model.* Cell Chem Biol, 2019. **26**(5): p. 623-633 e9.

328. Hu, K., et al., *Suppression of the SLC7A11/glutathione axis causes synthetic lethality in KRAS-mutant lung adenocarcinoma.* J Clin Invest, 2020. **130**(4): p. 1752-1766.

329. Scalise, M., et al., *The Human SLC1A5 (ASCT2) Amino Acid Transporter: From Function to Structure and Role in Cell Biology.* Front Cell Dev Biol, 2018. **6**: p. 96.

330. Cormerais, Y., et al., *The glutamine transporter ASCT2 (SLC1A5) promotes tumor growth independently of the amino acid transporter LAT1 (SLC7A5).* J Biol Chem, 2018. **293**(8): p. 2877-2887.

331. Luo, Y., et al., *ASCT2 overexpression is associated with poor survival of OSCC patients and ASCT2 knockdown inhibited growth of glutamine-addicted OSCC cells.* Cancer Med, 2020. **9**(10): p. 3489-3499.

332. Zhang, Z., et al., *ASCT2 (SLC1A5)-dependent glutamine uptake is involved in the progression of head and neck squamous cell carcinoma.* Br J Cancer, 2020. **122**(1): p. 82-93.

333. Zhang, H., et al., *Comprehensive molecular and clinical characterization of SLC1A5 in human cancers.* Pathol Res Pract, 2021. **224**: p. 153525.

334. Zhu, D., et al., *Ferroptosis-related gene SLC1A5 is a novel prognostic biomarker and correlates with immune infiltrates in stomach adenocarcinoma.* Cancer Cell Int, 2022. **22**(1): p. 124.

335. Zhao, J., et al., *Correlation Between Prognostic Biomarker SLC1A5 and Immune Infiltrates in Various Types of Cancers Including Hepatocellular Carcinoma.* Front Oncol, 2021. **11**: p. 608641.

336. Yoo, H.C., et al., *A Variant of SLC1A5 Is a Mitochondrial Glutamine Transporter for Metabolic Reprogramming in Cancer Cells.* Cell Metab, 2020. **31**(2): p. 267-283 e12.

337. Alves, M.J.F., et al., *The expression of the aminoacid transporters ASCT2 (SLC1A5) and LAT1 (SLC7A5) in astrocytomas.* Medical Express, 2016. **3**(6).

338. Sidoryk, M., et al., *Increased expression of a glutamine transporter SNAT3 is a marker of malignant gliomas.* Neuroreport, 2004. **15**(4): p. 575-8.

339. Esslinger, C.S., K.A. Cybulski, and J.F. Rhoderick, *Ngamma-aryl glutamine analogues as probes of the ASCT2 neutral amino acid transporter binding site.* Bioorg Med Chem, 2005. **13**(4): p. 1111-8.

340. Schulte, M.L., et al., *Pharmacological blockade of ASCT2-dependent glutamine transport leads to antitumor efficacy in preclinical models.* Nat Med, 2018. **24**(2): p. 194-202.

341. Ndaru, E., et al., *Novel alanine serine cysteine transporter 2 (ASCT2) inhibitors based on sulfonamide and sulfonic acid ester scaffolds.* J Gen Physiol, 2019. **151**(3): p. 357-368.

342. Garibsingh, R.A., et al., *Rational design of ASCT2 inhibitors using an integrated experimental-computational approach.* Proc Natl Acad Sci U S A, 2021. **118**(37).
